# A Degenerate Primer MOB Typing (DPMT) Method to Classify Gamma-Proteobacterial Plasmids in Clinical and Environmental Settings

**DOI:** 10.1371/journal.pone.0040438

**Published:** 2012-07-11

**Authors:** Andrés Alvarado, M. Pilar Garcillán-Barcia, Fernando de la Cruz

**Affiliations:** Departamento de Biología Molecular e Instituto de Biomedicina y Biotecnología de Cantabria, Universidad de Cantabria-Consejo Superior de Investigaciones Científicas-SODERCAN, Santander, Spain; Institut National de la Recherche Agronomique, France

## Abstract

Transmissible plasmids are responsible for the spread of genetic determinants, such as antibiotic resistance or virulence traits, causing a large ecological and epidemiological impact. Transmissible plasmids, either conjugative or mobilizable, have in common the presence of a relaxase gene. Relaxases were previously classified in six protein families according to their phylogeny. Degenerate primers hybridizing to coding sequences of conserved amino acid motifs were designed to amplify related relaxase genes from γ-Proteobacterial plasmids. Specificity and sensitivity of a selected set of 19 primer pairs were first tested using a collection of 33 reference relaxases, representing the diversity of γ-Proteobacterial plasmids. The validated set was then applied to the analysis of two plasmid collections obtained from clinical isolates. The relaxase screening method, which we call “Degenerate Primer MOB Typing” or DPMT, detected not only most known Inc/Rep groups, but also a plethora of plasmids not previously assigned to any Inc group or Rep-type.

## Introduction

Plasmids exert a great evolutionary impact in their bacterial hosts, allowing them to colonize new niches, obtain advantages against either natural competitors, or overcome artificial selective pressures. These beneficial characteristics easily spread between bacterial populations because of horizontal gene transfer. Among the clinically important disseminated traits are determinants for antibiotic resistance (AbR) and virulence [1], [2].

Basic physiological functions of plasmids are autonomous replication, stability and propagation (conjugation and establishment in new hosts) [3]. Differences in replication and stability constituted the basis for classifying plasmids, first by incompatibility (Inc) and later by replicon typing. Incompatibility (the inability of two plasmids to coexist within the same cell) is a phenotypic expression of the interactions in plasmid replication [4] or partition [5]. By Inc testing [6], enterobacterial plasmids were divided in 27 groups, with some further subdivisions [7]. Inc groups include historical R-plasmids, which largely contributed to AbR dissemination, together with xenobiotic biodegradation and virulence plasmids. The Inc classification did not always reflect true evolutionary divergence: highly similar plasmids can be compatible [8], [9], [10], [11], [12], [13], [14], while largely non homologous plasmids can be incompatible (*e.g.* IncX1 and IncX2 plasmids [15], [16], [17], some IncQ1 and IncQ2 plasmids [13]). As a consequence of the technical drawbacks of Inc testing, plasmid classification turned to molecular comparison of replication regions, leading to the development of two replicon typing methods. The first was based on DNA hybridization with specific plasmid probes (Inc/Rep-HYB) that contained either copy number control or partition DNA sequences of 19 Inc groups [18]. The second and presently most widely used method is called PCR-based replicon typing (PBRT). It was first used to identify five Inc groups of broad-host-range plasmids in environmental samples (IncW, IncP1, IncQ1, IncN [19], [20], [21] and IncP9 [22], [23]) and later on to detect replicons predominant in Enterobacteriaceae [24], [25], [26], [27], [28] as well as 19 groups of resistance plasmids of *Acinetobacter baumanii* [29]. Plasmid multilocus/double sequence type methods [27], [30], [31], [32], [33] and PCRs detecting plasmid genes other than replication/partition modules [19], [34] were also developed to detect some plasmid backbones. PBRT and these other methods allowed plasmid identification and circumvented the technical problems associated to Inc testing. As a drawback, they narrowed plasmid classification within the boundaries of Inc groups or small clusters of highly similar backbones. Thus, PBRT kept a significant fraction of plasmid groups out of assortment.

Around 50% of γ-Proteobacteria plasmids are potentially transmissible [35]. Conjugative plasmids encode all functions needed for transfer (*i.e.* origin of transfer locus (*oriT*), relaxase, coupling protein (T4CP) and type IV secretion system (T4SS)). Mobilizable plasmids code only for *oriT*, relaxase and nicking-accessory protein(s) (and only rarely for T4CP), requiring the help of a conjugative plasmid to be transferred. Thus, the only common component to all transmissible (conjugative and mobilizable) plasmids is the relaxase. Relaxases are multidomain proteins, the relaxase activity residing in their N-terminal domain [36]. The 3D structures of four relaxase domains have been solved: the MOB_F_ relaxases TrwC_R388 [37] and TraI_F [38], the MOB_Q_ relaxase MobA_R1162/RSF1010 [39] and the MOB_V_ relaxase MobM_pMV158 (M. Espinosa, personal communication). In these proteins, the architecture of the active centre is highly similar in spite of the fact that they belong to three different MOB families [35]. Homology at the sequence level resides on three conserved motifs: motif I that contains the catalytic Tyr residue(s) involved in DNA cleavage-joining reactions; motif II that contains an Asp or Glu residue involved in activation of the nucleophilic hydroxyl of the catalytic Tyr, and the most conspicuous motif III, which contains a His triad that coordinates a divalent cation directly involved in the catalytic reactions [37], [40]. The evolutionary relationships among relaxase sequences were traced and transmissible plasmids distributed in six relaxase MOB families [35], [36]. Here, we developed a set of oligonucleotide primers for relaxase identification based on the relaxase protein phylogenies. The method is called “Degenerate Primer MOB Typing” (DPMT). As an application, we used DPMT to identify new relaxases and to classify plasmids isolated from clinical isolates of γ-Proteobacteria.

## Results

### Design and Validation of the DPMT Oligonucleotide Set

**Figure 1:**
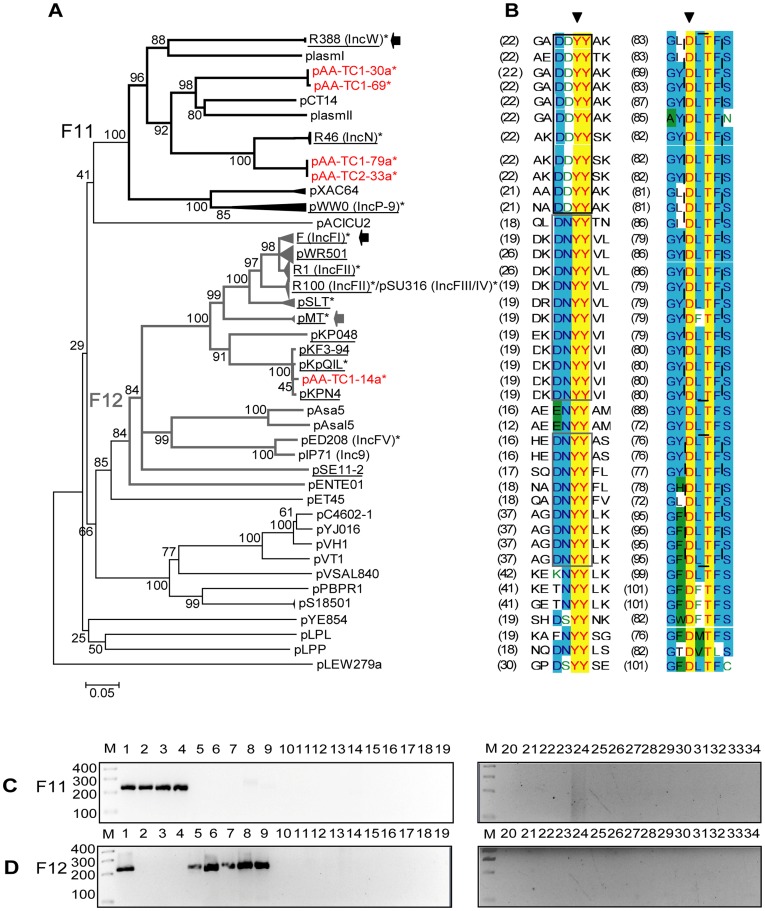
DPMT validation for MOB_F_ relaxases. A) Phylogenetic tree of MOB_F_ relaxases. Triangles at the end of the branches represent a compressed group of very similar relaxases (>95%). A solid black arrow points to the prototype plasmid for each subfamily. Arrows point to plasmids that experimentally amplified, in spite of containing at least one mismatch in the 12 nucleotides of the CORE sequence. Relaxases contained in our reference collection (Table 1) are denoted by an asterisk. Plasmids detectable by PBRT amplification [19], [20], [21], [22], [24], [25], [27], [28], [29] are underlined. New relaxase sequences uncovered by DPMT are shown in red. B) Alignment of the relaxase motifs used to design the MOB_F_ degenerate primers. Colour code: red on yellow  =  invariant amino acids; blue on blue  =  strongly conserved; black on green  =  similar; green on white  =  weakly similar; black on white  =  not conserved. Black arrowheads point to the key residues that define the relaxase motifs. Different rectangles embrace the conserved amino acids used to infer the 3′ degenerate core of each oligonucleotide (F11-f, continuous black; F12-f, continuous dark grey; and F1-r, dashed black). C) Amplicons obtained with primers for subfamily MOB_F11_ (F11-f and F1-r). Lane 1, pSU1588; 2, pSU4280; 3, pSU10013; 4, pSU10014; 5, pSU10017; 6, pSU10018; 7, pSU10021; 8, pSU316; 9, pSU10022; 10, pSU10010; 11, R751; 12, pSU10028; 13, pSU10029; 14, pSU10056; 15, pSU10055; 16, pSU10001; 17, pSU10012; 18, pSU10011; 19, pSU10009; 20, pSU4601; 21, pSU10006; 22, pSU10007; 23, pSU10064; 24, pSU10059; 25, pSU10008; 26, pSU10039; 27, pSU10040; 28, pSU10041; 29, pSU10004; 30, pSU10003; 31, pSU10043; 32, pSU4830; 33, pSU10002; 34, negative control. Lane M, molecular mass marker, HyperLadder IV (Bioline). D) Amplicons obtained with primers for subfamily MOB_F12_ (F12-f and F1-r). Lanes as in (C).

**Figure 2 pone-0040438-g002:**
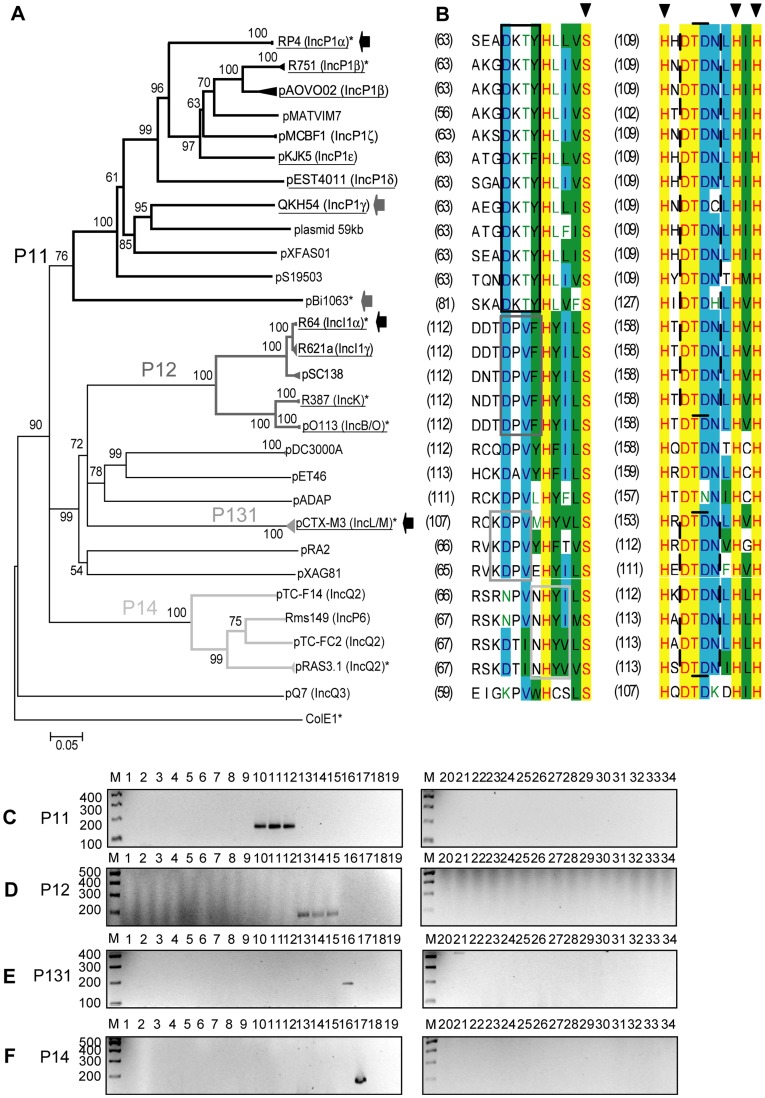
DPMT validation for MOB_P1_ relaxases. A) Phylogenetic tree of MOB_P1_ relaxases. B) Alignment of the relaxase motifs used to design the MOB_P1_ degenerate primers (P11-f, continuous black; P12-f, continuous dark grey; P131-f, continuous grey; P14-f, continuous light grey; and P1-r, dashed black). C) Amplicons obtained with primers for subfamily MOB_P11_ (P11-f and P1-r). D) Amplicons obtained with primers for subfamily MOB_P12_ (P12-f and P1-r). E) Amplicons obtained with primers for subfamily MOB_P13_ (P131-f and P1-r). F) Amplicons obtained with primers for subfamily MOB_P14_ (P14-f and P1-r). Symbols, colour codes and lanes as in Figure 1.

**Figure 3 pone-0040438-g003:**
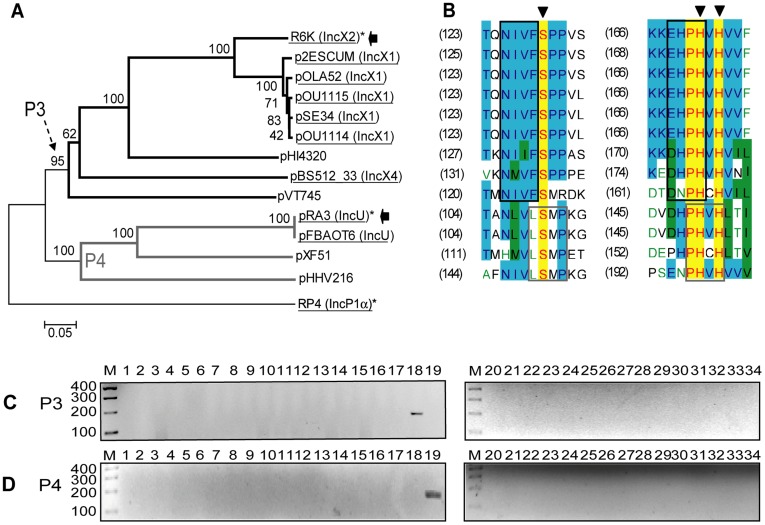
DPMT validation for MOB_P3_ and MOB_P4_ relaxases. A) Phylogenetic tree of MOB_P3_ and MOB_P4_ relaxase families. B) Alignment of the relaxase motifs used to design the MOB_P3_ and MOB_P4_ degenerate primers (P3-f+P3-r, continuous black; and P4-f+P4-r, continuous dark grey). C) Amplicons obtained with primers for subfamily MOB_P31_ (P3-f and P3-r). D) Amplicons obtained with primers for subfamily MOB_P42_ (P4-f and P4-r). Symbols, colour codes and lanes as in Figure 1.

**Figure 4 pone-0040438-g004:**
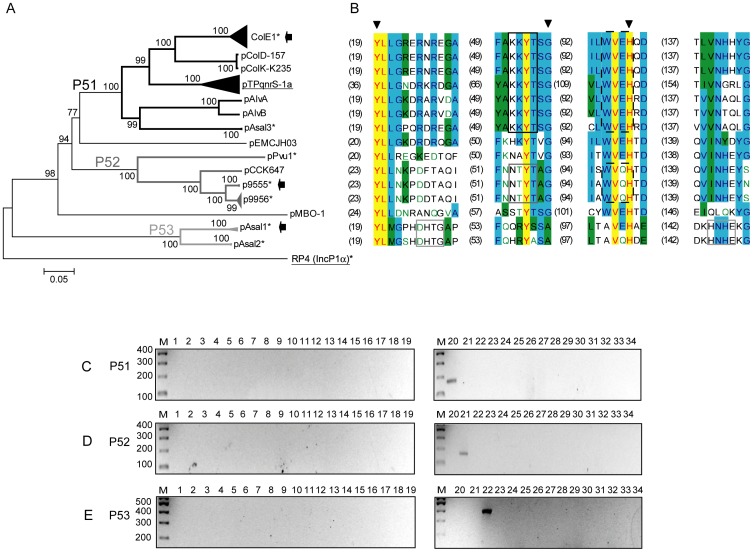
DPMT validation for MOB_P5_ relaxases. A) Phylogenetic tree of MOB_P5_ relaxase family. B) Alignment of the relaxase motifs used to design the MOB_P5_ degenerate primers (P51-f, continuous black; P52-f, continuous dark grey; P5-r, dashed black; and P53-f+P53-r, continuous grey) C) Amplicons obtained with primers for subfamily MOB_P51_ (P51-f and P5-r). D) Amplicons obtained with primers for subfamily MOB_P52_ (P52-f and P5-r). E) Amplicons obtained with primers for subfamily MOB_P53_ (P53-f and P53-r). Symbols, colour codes and lanes as in Figure 1.

**Figure 5 pone-0040438-g005:**
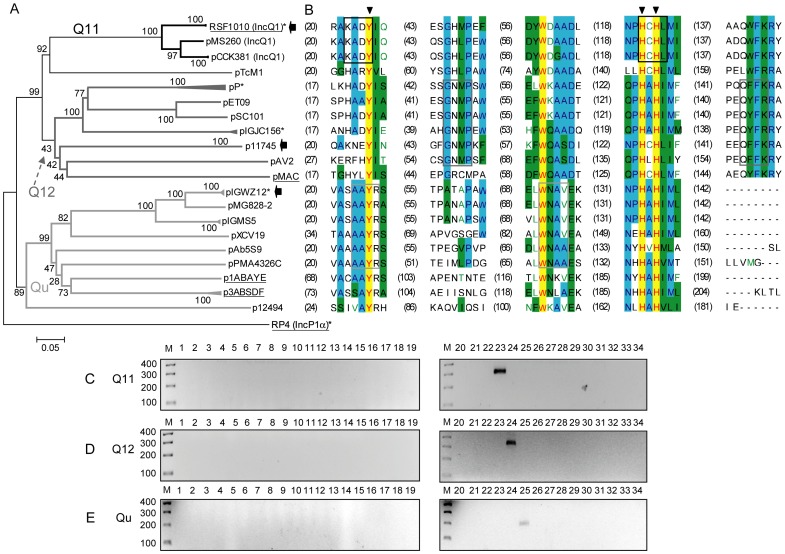
DPMT validation for MOB_Q_ relaxases. A) Phylogenetic tree of MOB_Q_ relaxase family. B) Alignment of the relaxase motifs used to design the MOB_Q_ degenerate primers (Q11-f+Q11-r, continuous black; Q12-f+Q12-r, continuous dark grey; and Qu-f+Qu-r, continuous grey). C) Amplicons obtained with primers for subfamily MOB_Q11_ (Q11-f and Q11-r). D) Amplicons obtained with primers for subfamily MOB_Q12_ (Q12-f and Q12-r). E) Amplicons obtained with primers for subfamily MOB_Qu_ (Qu-f and Qu-r). Symbols, colour codes and lanes as in Figure 1.

**Figure 6 pone-0040438-g006:**
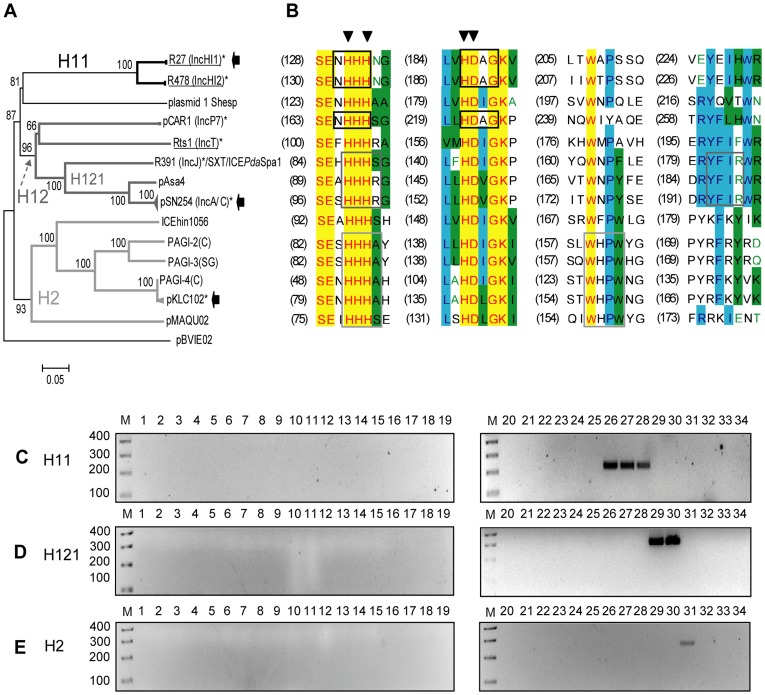
DPMT validation for MOB_H_ relaxases. A) Phylogenetic tree of MOB_H_ relaxase family. B) Alignment of the relaxase motifs used to design the MOB_H_ degenerate primers (H11-f+H11-r, continuous black; H121-f+H121-r, continuous dark grey; and H2-f+H2-r, continuous grey). C) Amplicons obtained with primers for subfamily MOB_H11_ (H11-f and H11-r). D) Amplicons obtained with primers for subfamily MOB_H121_ (H121-f and H121-r). E) Amplicons obtained with primers for subfamily MOB_H2_ (H2-f and H2-r). Symbols, colour codes and lanes as in Figure 1.

**Figure 7:**
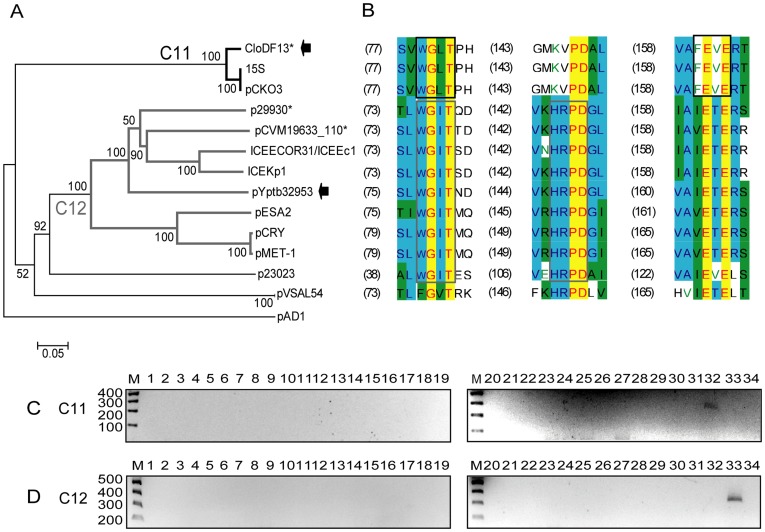
DPMT validation for MOB_C_ relaxases. A) Phylogenetic tree of MOB_C_ relaxase family. B) Alignment of the relaxase motifs used to design the MOB_C_ degenerate primers (C11-f+C11-r, continuous black; C12-f+C12-r, continuous dark grey). C) Amplicons obtained with primers for subfamily MOB_C11_ (C11-f and C11-r). D) Amplicons obtained with primers for subfamily MOB_C12_ (C12-f and C12-r). Symbols, colour codes and lanes as in Figure 1.

Phylogenetic trees of the five plasmid relaxase families which contained suitably populated and well supported subfamilies in γ-Proteobacteria were traced as shown in Figures 1, 2, 3, 4, 5, 6, 7. They served as guides for designing oligonucleotide primer pairs able to amplify relaxases clustered in those subfamilies. Each primer was partially degenerated, up to 24 degeneracy at its 3′ sequence, to encompass a relaxed codon usage. Primers for which the design resulted in degeneracy larger than 24, were reduced to degeneracy-24 by considering only the sequences present in the respective DNA relaxase alignment. Each primer pair was tested on a reference collection of 33 relaxases encoded by transmissible plasmids originally isolated from γ-Proteobacteria (Table 1). Once their specificity was validated, the set of validated primers was used to identify relaxases in plasmid collections from clinical isolates, leading to the identification of both known and non-previously reported relaxase sequences. Details for the design and range of substrates of the primer pairs selected for each MOB family follow.

**Table 1:** Plasmids and relaxase genes used as controls in validation experiments.

*Plasmid*	*Cloned gene*	*Plasmid accession number* a	*Position* b	*Inc Group^3^*	*MOB Subfamily^4^*	*Reference*
pSU1588	trwC_R388	BR000038	14128–15007*	IncW	F11	[79]
pSU4280	pKM101 complete MOB region	U09868	14810–20208*	IncN	F11	[80]
pSU10013	traC_pBi709	AY299015	17902–18771*	–	F11	This study
pSU10014	traC_Pwwo	NC_003350	98516–99385	IncP-9	F11	This study
pSU10017	traI_F	NC_002483	92673–93590	IncFI	F12	This study
pSU10018	traI_R100	NC_002134	78466–79401	IncFII	F12	This study
pSU10021	traI_pSLT	NC_003277	87282–88199	IncFII	F12	This study
pSU316	–	M26937, X55894, M28097	–	IncFIII-IncFIV	F12	[81]
pSU10022	traI_pED208	AF411480	25650–26552	IncFV	F12	This study
pSU10010	traI_RP4	X54459	3389–4198*	IncP-1α	P11	This study
R751	–	NC_001735	–	IncP-1β	P11	
pSU10028	traI_pBI1063	AY299014	3848–4675*	–	P11	This study
pSU10029	nikB_R64	NC_005014	67391–68350	IncI1	P12	This study
pSU10056	nikB_ R387	M93063, X07848	–	IncK	P12	This study
pSU10055	nikB_pO113	NC_007365	62419–63393*	IncB/O	P12	This study
pSU10001	nikB_pCTX-M3	NC_004464	32027–33049	IncL/M	P131	This study
pSU10012	mobA_pRAS3.1	NC_003123	10571–11395*	IncQ2	P14	This study
pSU10011	taxC_R6K	Y10906, X95535	–	IncX2	P31	This study
pSU10009	nic_pRA3	NC_010919	10360–11355	IncU	P42	This study
pSU4601	ColE1::kan	NC_001371	–	ColE1	P51	[82]
pSU10006	mobA_p9555	NC_010069	3368–4394	–	P52	This study
pSU10007	mobA_pAsal1	NC_004338	1052–2017	–	P53	This study
pSU10064	mobA_RSF1010	NC_001740	3250–3807	IncQ1	Q11	This study
pSU10059	ORF1_pP	NC_003455	9–1244	–	Q12	This study
pSU10008	mobA/mobL_pIGWZ12	NC_010885	1257–2240*	–	Qu	This study
pSU10039	traI_R27	NC_002305	106098–106934*	IncHI1	H11	This study
pSU10040	traI_R478	NC_005211	192385–193308	IncHI2	H11	This study
pSU10041	traI_pCAR1	NC_004444	124079–125008	IncP-7	H11	This study
pSU10004	traI_pSN254	NC_009140	46409–47593	IncA/C	H121	This study
pSU10003	traI_R391	AY090559	32341–33509	IncJ	H121	This study
pSU10043	traI_2_pKLC102	AY257538	99952–100788	–	H2	This study
pSU4830	mobC_CloDF13	NC_002119	–	–	C11	[83]
pSU10002	triL_p29930	AJ519722	31361–32107	–	C12	This study

a Accession number of the transmissible plasmid encoding the corresponding relaxase gene.

b Nucleotide coordinates of the cloned relaxase fragment in the accession number of the original plasmid. An asterisk indicates that the relaxase gene is coded in the complementary strand of the original plasmid sequence.

c Incompatibility group of the wt plasmid.

d MOB subfamily of each relaxase gene.

#### MOB_F_ family

Figure 1A shows the phylogenetic reconstruction of MOB_F_ relaxases from γ-proteobacterial plasmids. Two subfamilies contain most MOB_F_ relaxases found in clinically relevant plasmids. Subfamily MOB_F11_ includes, among others, relaxases of AbR plasmids from Inc groups W, N as well as metal-resistance and xenobiotic-biodegradation plasmids of Pseudomonas group IncP-9. Subfamily MOB_F12_ contains relaxases of AbR and virulence plasmids of the IncF complex (IncFI, IncFII, IncFIII and IncFV) and Inc9 (also known as com9), widely distributed among different genera of Enterobacteriaceae. Specific amplification of MOB_F11_ and MOB_F12_ plasmids was obtained with two forward primers (*F11-f* and *F12-f*) and one reverse primer (*F1-r*) (Table 2, Figure 1B–D). Since both forward primers differ only by a single nucleotide, cross-amplification was occasionally observed between MOB_F11_ and MOB_F12_ relaxases. Thus, the two amplification reactions identified the most relevant MOB_F_ plasmids but did not discriminate among them.

**Table 2:** List of degenerate primers used for DPMT.

*Primer* *name*	*Primer sequence* a	*PCR conditions*	*Prototype* b	*Amplicon* *size (bp)* c	*Amplicon* *location* d
*F11-f*	gca gcg tat tac ttc tct gct gcc **gay gay tay ta**	25 cycles, 53°C	R388	234	13876–14047*
*F1-r*	act ttt ggg cgc gga **raa btg sag rtc**				
*F12-f*	agc gac ggc aat tat tac acc gac aag **gay aay tay ta**	25 cycles, 55°C	F	234	92744–92912
*F1-r*	act ttt ggg cgc gga **raa btg sag rtc**				
*P11-f*	cgt gcg aag ggc gac **aar acb tay ca**	25 cycles, 60°C	RP4	180	50361–50484*
*P1-r*	agc gat gtg gat gtg aag **gtt rtc ngt rtc**				
*P12-f*	gca cac tat gca aaa gat gat act **gay ccy gtt tt**	30 cycles, 53.8°C, 1.5U Taq per reaction	R64	189	67744–67867
*P1-r*	agc gat gtg gat gtg aag **gtt rtc ngt rtc**				
*P131-f*	aac cca cgc tgc **aar gay ccv gt**	30 cycles, 59°C, 15 seconds of extension per cycle	pCTX-M3	180	32365–32491
*P1-r*	agc gat gtg gat gtg aag **gtt rtc ngt rtc**				
*P14-f*	cgc agc aag gac acc atc **aay cay tay** **rt**	25 cycles, 50°C	pRAS3.1	174	11053–11169*
*P1-r*	agc gat gtg gat gtg aag **gtt rtc ngt rtc**				
*P3-f*	cc gtg agc caa atc aca cag **aat atk rtb tt**	25 cycles, 50°C	R6K	177	38419–39573*
*P3-r*	cg aaa gcc aac atg aac **atg hgg atk htc**				
*P4-f*	gcg ttc agg atg gtc **ytb tcs atg cc**	25 cycles, 64°C	pRA3	163	10695–10803
*P4-r*	c ggt ttt gac cgt cag **atg svm atg cgg**				
*P51-f*	t acc acg ccc tat gcg **aar aar tay ac**	30 cycles, 58°C, 20 seconds of extension per cycle	ColE1	167	572–688
*P5-r*	cc ctt gtc ctg **gtg yts nac cca**				
*P52-f*	gat agc ctt gat ttt aat aac acc **aay acy tay ac**	30 cycles, 58°C, 20 seconds of extension per cycle	p9555	175	3536–3652
*P5-r*	cc ctt gtc ctg **gtg yts nac cca**				
*P53-f*	g ggc tcg cac **gay cay acn gg**	30 cycles, 65°C	pAsal1	345	1136–1480
*P53-r*	gc cca gcc ctt **ttc rtg rtt rtg**				
*Q11-f*	caa tcg tcc aag gcg **aar gcn gay ta**	30 cycles, 50°C	RSF1010	331	3325–3606
*Q11-r*	cg ctc gga gat cat **cay ytg yca ytg**				
*Q12-f*	ctg gaa tat act gaa cac **ggn aay atg cc**	30 cycles, 52°C	pP	341	975–1256
*Q12-r*	atc ctt ggt gtt agc acg **ttt raa rwa ytg**				
*Qu-f*	agc gcc gtg ctg tcc **gcb gcn tay cg**	30 cycles, 64°C	pIGWZ12	179	2034–2162*
*Qu-r*	ctc cgc agc ctc **grc sgc rtt cca**				
*H11-f*	ccg gcg tcg gag **aay cay cay ca**	Touchdown PCR: start at 65°C ΔTa = −1°C per cycle, 15 cycles at 55°C	R27	207	106380–106536
*H11-r*	aag gtc gta tac ctt **ycc kgc rtc rtg**				
*H121-f*	g cca gct tcc gaa tca **cay cay cay cg**	25 cycles, 59°C	pSN254	313	46714–46981
*H121-r*	g tcg ctt gtc gcg cca **ccg dat raa rta**				
*H2-f*	ag ttc cca gcc tca gaa atc **cay cay cay kc**	25 cycles, 68°C	pKLC102	264	100218–100428
*H2-r*	g cgg acc gtg **cca ngg rtg cca**				
*C11-f*	gt cag gtc agc gtg **tgg ggn ctn ac**	Touchdown PCR: start at 65°C ΔTa = −1°C per cycle, 20 cycles at 55°C	CloDF13	283	2874–3106
*C11-r*	ct ctt cac ggt gcc **ctc nac ytc raa**				
*C12-f*	gc acg act gga aaa ata tcg cta **tgg ggn ath ac**	30 cycles, 59°	p29930	257	31594–31789
*C12-r*	caa cgt gat aat ccc **gtc rgg vcg rtg**				

a For each oligonucleotide, CORE nucleotides are in bold and CLAMP sequences in normal lettering. Underlined codons do not encompass all the possible variability to avoid excessive degeneracy. The sequences used are biased to accommodate the DNA sequences of existing elements.

b Prototype plasmid for the given MOB subfamily.

c Amplicon size obtained from the prototype plasmid relaxase gene.

d Nucleotide coordinates of the prototype plasmid contained in the corresponding amplicon. An asterisk indicates that the relaxase gene is encoded in the complementary strand.

#### MOB_P_ family

Within γ-Proteobacteria, MOB_P_ contains relaxases of AbR plasmids belonging to the IncP1 complex (IncP1α, IncP1β, IncP1δ, IncP1γ, IncP1ε, and IncP1ζ), many of them recovered from soil and manure isolates [21], virulence and AbR plasmids of the IncI complex (IncI1α, IncI1γ, IncK, IncB/O), AbR plasmids IncL/M, IncQ2 (IncQ2α, IncQ2β, IncG/IncP-6, IncX1, IncX2, IncU and IncQ3 groups, plus several other branches that contained no Inc prototype. The ample diversity of this family was reflected in the MOB_P_ phylogeny, which showed several well-resolved monophyletic groups, as well as additional, poorly-defined deep branches [35]. Thus, to construct the set of MOB_P_ primers we had to manage each subfamily separately. Relaxases of IncP1α, IncP1β, IncP1δ, IncP1γ, IncP1ε, IncP1ζ, IncI1α, IncI1γ, IncK, IncB/O, IncL/M, IncQ2α, IncQ2β and IncG/IncP-6 plasmids -among others without Inc assignment- are grouped in the MOB_P1_ subgroup (Figure 2A); those of IncX1 and IncX2 plasmids are in group MOB_P3_ (Figure 3A); IncU plasmid relaxases are in group MOB_P4_ (Figure 3A), and relaxases of ColE1-related plasmids in MOB_P5_ (Figure 4A). Neither subfamily MOB_P6_, which contains a scarce number of γ-Proteobacteria relaxases (including those in IncI2 plasmids), nor other poorly resolved clades (as the one containing IncQ3 plasmids), were considered in this study.

##### MOB_P1_ subfamily

One reverse and four forward primers were needed for amplification of MOB_P1_ relaxases (Figure 2B, Table 2). The *P11-f* forward primer led to amplification of MOB_P11_ plasmids (including IncP1). Similarly, the *P12-f* forward primer identified MOB_P12_ plasmids (including IncI1, IncK, and IncB/O), *P131-f* forward primer identified MOB_P13_ plasmids (including IncL/M), and *P14-f* forward primer identified MOB_P14_ plasmids (including IncQ2 and IncG). Results are shown in Figure 2C–F. No cross-amplification was observed, except for *P131-f* + *P1-r* when using plasmid p9555 as template (Figure 2E). The non-specific amplicon was larger than that obtained from the reference MOB_P131_ relaxase gene *nikB*_pCTX-M3, so the interpretation of the data was unambiguous.

##### MOB_P3_ and MOB_P4_ subfamilies

MOB_P3_ relaxases correspond to IncX1 and IncX2 plasmids while MOB_P4_ contains relaxases of IncU plasmids (Figure 3A and Table S1). One primer pair was designed for each subfamily. No cross-amplification was observed (Figure 3C–D), except for the fortuitous amplification of some *Salmonella* chromosomes described in Methods, subsection “Validation and methodologies comparison”.

##### MOB_P5_ subfamily

Most MOB_P5_ (ColE1-like) relaxases lack the canonical 3H motif III, but contain a deviant HEN motif [41] (Figure 4B). Three primer pairs (*P51*, *P52* and *P53*, Table 2) were designed to amplify this cluster (Figure 4), two pairs specific for plasmids with a HEN motif (*P51* and *P52*) and one for plasmids with the 3H motif (*P53*).

#### MOB_Q_ family

Phylogenetic reconstruction of γ-proteobacterial MOB_Q_ relaxases showed two distinguishable MOB_Q_ clades, MOB_Q1_ and MOB_Qu_ (Figure 5A). For amplifying the first broad clade, two primer pairs were designed, *Q11* and *Q12*, and one primer pair, *Qu*, for the MOB_Qu_ cluster (Figure 5B, Table 2). Some phylogenetic overlapping between MOB_Q_ and MOB_P_ families has been reported [35]. Nevertheless, primers that hit each relaxase branch did not cross-amplify (Figure 5C–E).

#### MOB_H_ family

MOB_H_ relaxases are encoded by AbR IncHI1, IncHI2, IncA/C, IncT, and xenobiotic-biodegradation Pseudomonas P7 plasmids, as well as by some ICEs (*e.g.* R391/SXT-like, *clc*, PAPI-1, etc.) (Figure 6A, Table S1). R391-like elements, exhibiting incompatibility properties, were formerly considered as plasmids and classified as IncJ [42], [43]. MOB_H_ relaxases have, besides the canonical conserved regions, additional motifs related to HD-hydrolases [44]. Three primer pairs were used to amplify MOB_H_ relaxases (Figure 6C–D, Table 2): *H11* (specific for IncHI1, IncHI2 and P-7 plasmids, represented by R27, R478 and pCAR1 respectively), *H121* (amplifying IncA/C and R391-like elements, represented by pSN254 and R391 respectively) and *H2* (amplifying a large set of relaxases from a family of ICEs, like pKLC102).

#### MOB_C_ family

All MOB_C_ relaxases encoded in γ-proteobacterial plasmids cluster in a single clade, MOB_C1_, when outgrouping with Firmicutes/Tenericutes MOB_C_ relaxases (Figure 7A, Table S1). MOB_C_ relaxases present in ICEs, such as ICE*Kp1* and ICE*Ec1* also cluster in clade C1. MOB_C_ is a peculiar relaxase family that does not contain the three classical signature motifs present in all other MOB families. Two primer pairs were designed to amplify each MOB_C1_ subclade: *C11* and *C12* (Figure 7 B–D, Table 2).

### Analysis of Clinical Plasmid Collections Using DPMT

Once validated by testing the reference collection of relaxases (Table 1), the set of 19 primer pairs was used to screen two plasmid collections from clinical samples as test cases (Table 3).

**Table 3:** Relaxases found in two test collections.

*Test collection* a	*MOB_F_*		*MOB_P_*									*MOB_Q_*			*MOB_H_*			*MOB_C_*		*Total*
	F11	F12	P11	P12	P13	P14	P3	P4	P51	P52	P53	Q11	Q12	Qu	H11	H121	H2	C11	C12	
1	14	60	4	39	6	0	3	0	71	0	0	0	5	11	13	10	0	0	1	237
2	0	30	2	6	0	0	7	0	18	0	0	0	0	4	1	0	0	3	6	77
*Total*	14	90	6	45	6	0	10	0	89	0	0	0	5	15	14	10	0	3	7	314

a Isolate collections analyzed with DPMT.

Test collection 1 consisted of 135 isolates of Enterobacteriaceae, recovered in different countries (Canada, Portugal, Spain, France and Kuwait) from 1989 to 2008, and producing extended spectrum beta-lactamases (ESBL). 104 of them were *E. coli* transconjugants harbouring ESBL-coding plasmids from different Enterobacteriaceae donors while the remaining 31 were original donors unable to conjugate the ESBL determinant. The collection mainly included plasmid-encoded ESBLs from class A (SHV (4/135; [45], TEM (18/135; [46], [47], [48]) and CTX types (91/135; [45], [46], [47], [49], [50], [51], [52]). A total of 237 relaxases were identified in the 135 strains, distributed among the five MOB families targeted by the primer set. The resulting amplicons were sequenced. Out of 237 sequenced amplicons, only five corresponded to relaxase sequences not previously reported (we consider a relaxase new when it shows less than 95% amino acid sequence identity with the closest hit in the NCBI nr database). Two of them, corresponding to plasmids pAA-TC1-69 and pAA-TC1-30a (GenBank Accession numbers JN167247 and JN167248), respectively exhibited 62% and 64% amino acid identity to the MOB_F11_ relaxase of plasmid pCT14 (nearest hit). Two others, those of plasmids pAA-TC1-79a and pAA-TC2-33a, were 78% identical to R46 relaxase (details in Information S1), suggesting overall more diversity within the MOB_F11_ relaxase branch than anticipated from the analysis of present genome databases. Complete sequencing of the relaxase domain of these plasmid genes and the ensuing phylogenetic analysis classified them as well defined new branches in the MOB_F11_ phylogeny (incorporated to Figure 1 in red color). Similarly, a fifth relaxase, that of plasmid pAA-TC1-14a, was 87% identical to pKPN4 relaxase and was classified as MOB_F12_ (see Information S1). The finding of these five new relaxase sequences underscores the potency of DPMT to detect and classify plasmids unidentifiable by PBRT. The most represented MOB subfamilies in Test Collection 1 were MOB_P5_ (71 relaxases), MOB_F12_ (60), and MOB_P12_ (39), followed by MOB_H_ (23), MOB_Q_ (16) and MOB_F11_ (14). Finally, 7 out of 135 isolates, corresponding to transconjugants, did not render any relaxase amplicon. Since they probably code for relaxases of MOB subfamilies not considered in this work or new deviant relaxases, they were selected for complete sequencing and further investigation (work in progress).

Test collection 2 comprised *E. coli* isolates from urine cultures of Swedish women who suffered from uncomplicated, community-acquired urinary tract infections treated with pivmecillinam [53]. The isolates were assorted according to their PFGE profiles (Ellen Zechner, personal communication). We analyzed 49 representative isolates for the presence of relaxases using the same set of 19 MOB primer pairs. 30 out of the 49 primary strains gave positive amplification with at least one primer pair. The 19 isolates without positive DPMT results were used as donors in mating experiments. Transconjugants were obtained for 18 of them by using a battery of antibiotic resistances matching the donor AbR profiles. Selected transconjugants were tested again with the same set of primers. 13 out of 18 rendered amplicons with at least one primer pair, while five transconjugants remained unidentifiable. A total of 77 relaxase amplicons were obtained from the collection. 50 of them were sequenced, from which two corresponded to non-previously reported relaxase sequences; one MOB_P12_, pAA-A3201, was 80% identical to pO113 relaxase; and one MOB_Qu_, pAA-A3488, was 72% identical to pSMS35_4 relaxase (see Information S1). Finally, a third relaxase, pAA-A3180 (Accession number JN167246), showed 97% amino acid identity to MOB_F12_ plasmid R1 relaxase. In summary, the analysis of this second collection identified two new relaxase sequences, representing in turn new branches in the MOB family trees. The most abundant MOB family was MOB_P_ with 31 relaxases (18 belonging to subfamily MOB_P5_, 7 to MOB_P3_ and 6 to MOB_P12_), followed by MOB_F_, with 30 amplicons, all members of subfamily MOB_F12_. It is worth mentioning that the identification of 4 MOB_Qu_ and 9 MOB_C_ plasmids of this collection would have not been possible by using the available PBRT or Inc/Rep-HYB probes.

## Discussion

**Figure 8:**
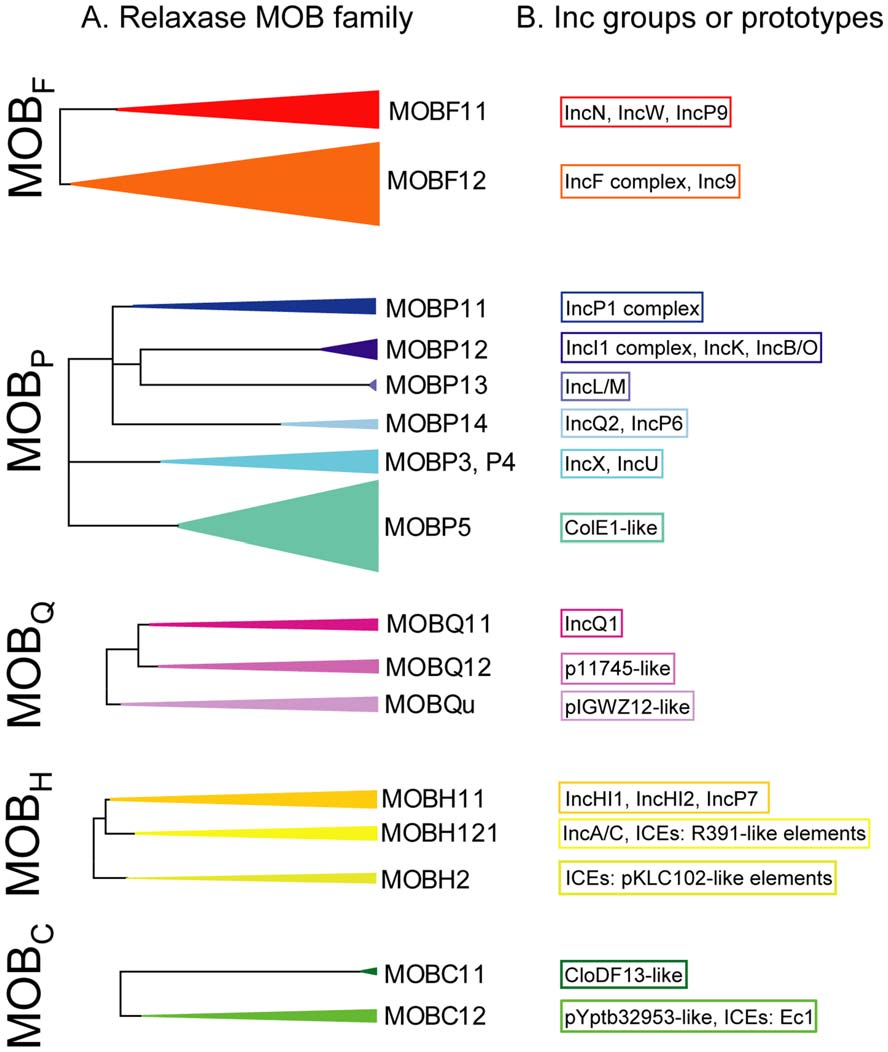
Correspondence between MOB and Rep types. A) Simplified phylogenetic representation of the five relaxase MOB families considered in this study. Coloured triangles represent the MOB subfamilies amplified by DPTM. Their width and depth correspond, respectively, to the abundance and phylogenetic diversity of their relaxase sequences (Table S1). B) The Inc groups contained within each MOB subfamily are indicated at the right, boxed in the same colour. When no Inc group is contained, the name of a prototype plasmid is given.

PBRT typing methods significantly improved the assignment of plasmids to Inc groups without the need to test for plasmid incompatibility despite some drawbacks like cross-hybridization between members of closely related Inc groups (such as IncI, IncK and IncB/O [18], [24]), false negative PCR results obtained when classifying more divergent plasmid groups (*e.g.* IncL/M [24]), and poor coverage of some groups (*e.g.* IncA/C [54], and ColE1-like [25]). PBRT identifies plasmids that belong to well-defined Inc groups. Nevertheless, a relevant part of the existing plasmid diversity, found in different ecological niches [55], [56], [57], [58], [59] that includes clinical settings [60], [61], remains elusive to PBRT classification (see Figure 8). In order to capture a broader range of plasmids, we considered groups of evolutionary related plasmid sequences instead of focussing on single sequences as PBRT usually does. Therefore, our set of primer pairs was not mainly designed to be used for screening purposes, but for the discovery of new relaxases and thus to expand and better delimit the known MOB subfamilies.

For the purpose of discovering new relaxases, we took advantage of the phylogenetic studies carried out with relaxase sequences [35], [36], [62], [63]. According to their relaxase genes, plasmids belong to six MOB families: MOB_F_, MOB_P_, MOB_Q,_ MOB_H_, MOB_C_, and MOB_V_ [35], [62]. Within each MOB family, the different taxa form groups with high amino acid sequence identity that allow us to define robust phylogenetic branches. Due to the clinical and epidemiological importance of γ-Proteobacterial plasmids, the phylogenies of relaxase subfamilies of plasmids hosted in γ-Proteobacteria were updated for this work (Figures 1 to [Fig pone-0040438-g002] [Fig pone-0040438-g003] [Fig pone-0040438-g004] [Fig pone-0040438-g005] [Fig pone-0040438-g006] 7). All in all, 17 subfamilies contained most of the diversity found in γ-Proteobacterial plasmids (summarized in Figure 8): subfamilies F11 and F12 from MOB_F_ (Figure 1); P11, P12, P13, P14, P3, P4 and P5 from MOB_P_ (Figures 2, 3, 4); Q11, Q12 and Qu from MOB_Q_ (Figure 5); H11, H12, and H2 from MOB_H_ (Figure 6); and C11 and C12 from MOB_C_ (Figure 7). Plasmids detected by PBRT are almost always included in these subfamilies (correspondences in Figure 8). The only exceptions are groups known not to contain relaxases (IncR, GR1/GR12, GR2/GR10, GR13, GR14, and GR17, whose prototype plasmids are pK245, pABSDF, pACICU1, p3ABAYE, p4ABAYE, and pAB1, respectively) or groups which do not contain any fully sequenced member or no relaxase in the known sequences (IncY (plasmid P1), IncFVI (pSU212), IncFVII (pSU221), GR3 (p736), GR4 (p844), GR5 (p537), GR8 (p11921), and GR16 (pAB49)). Families and subfamilies that contain only a few plasmid relaxases from γ-Proteobacteria, such as MOB_V_, MOB_P6_ (containing IncI2 plasmids), and some other poorly resolved clades (*e.g.* IncT and IncQ3 plasmids), were not considered in this study.

A computational protocol to search for conjugative and mobilizable genetic modules in a set of 1,730 completely sequenced plasmids recorded in the NCBI database, detected a relaxase in 260 out of the 503 plasmids hosted in γ-Proteobacteria [62]. We used that plasmid set to compare the detection capabilities of the available PBRT and DPMT probes (Table S1). Our set of 19 degenerate primer pairs was potentially able to detect 193 out of the 271 relaxases contained in the 260 transmissible γ-proteobacterial plasmids, that is, it would allow the classification of 186 out of these plasmids. Available PBRT probes (58 primer pairs) could potentially detect 153 plasmids in the total set, of which 98 were contained in the transmissible plasmid set. 87 out of 260 transmissible plasmids could be potentially detected by both PBRT and DPMT probes. This comparison suggests that DPMT is a powerful tool to detect and phylogenetically classify γ-proteobacterial transmissible plasmids.

A reference collection of 33 relaxases, containing representatives of the main MOB subfamilies, was used to test for specific amplification of the chosen primer pairs (Table 1). With few exceptions (see sections MOB_F_ family and MOB_P1_ subfamily), no cross-amplification between MOB subfamilies was observed. Several DPMT primer pairs have already been successfully used conjointly with PBRT for identifying plasmids from clinical strains [47], [64], [65]. In this work we analyzed two enterobacterial plasmid collections by DPMT, capturing not only the known Inc plasmid groups but also a number of others undetected by PBRT, some of which contained new relaxase sequences. The DPMT method only failed to identify a MOB relaxase in 12 out of 122 transconjugants from these collections. Failure to find a relaxase in an experimentally verified transconjugant could be attributed to: i) the sequence bias introduced in some primers to avoid high degeneracy (see Table 2), ii) the presence of relaxases belonging to subfamilies not included as targets by our primer set, or iii) the existence of relaxases whose sequences could be largely deviant from the subfamily consensus. In any case, the results presented in this work suggest that the present implementation of the DPMT method identifies more than 90% of the transmissible R-plasmids in transconjugants of clinical isolates. Once less-populated or poorly-resolved relaxase phylogenetic clades become more robust by accretion of further data, our method could be expanded to allow the identification of a higher proportion of relaxases. Our ongoing work aims to do so, with the collaboration of a number of clinical research groups in Spain and Europe.

Detection of transmissible plasmids by PBRT and DPMT underscores their complementarities in focus and scope. While PBRT focuses in replication or partition regions shared by clusters of highly-related plasmids (>95% nucleotide identity), DPMT targets relaxase motifs conserved in large groups of plasmids with deep phylogenetic diversity. As shown in Results, we can detect relaxases with as little as 60% amino acid sequence identity to the nearest known hit in the databases. Thus, PBRT is useful at detecting blooms of redundant backbones that carry different cargo genes (“zoom in” strategy), while DPMT finds and classifies backbones that share a common relaxase ancestor (“zoom out” strategy). Most PBRT primers were designed for detecting plasmids from Enterobacteriaceae [24], [27], although there are a few available for detection of plasmids from other taxonomic families of γ-Proteobacteria, such as IncP-1 [19], [20], [21], IncP-9 [22], [23], or *Acinetobacter baumannii* replicons [29]. The vast diversity in the plasmid world makes the design of probes that target small groups of highly-related plasmids a strategy limited in practical terms for specific purposes, not suitable for studying global diversity neither for finding deviant plasmids from well-studied backbones. The DPMT strategy is more inclusive, allowing the detection of plasmids hosted by a larger number of taxonomic families. Nevertheless, it should be emphasized that it still recovers a higher proportion of plasmids from Enterobacteriaceae (85%) than from other γ-Proteobacterial families (51.4%). This is mostly due to the lack of a suitable number of related relaxase sequences to construct robust phylogenetic trees, as exemplified, for instance, by the Moraxellaceae, Vibrionaceae, Pseudomonadaceae and Aeromonadaceae plasmids [35], [62]. Perhaps investigators in public health surveillance, veterinary or environmental science should consider the interest of developing sets of oligonucleotide pairs more specifically adapted to their needs. Most clinically relevant transmissible plasmids detected by PBRT probes are also uncovered by DPMT, as shown in this work. On the contrary, no PBRT probes are available for many plasmids detected by DPMT such as the virulence plasmids IncFIII/IV (MOB_F12_), IncQ2 (MOB_P14_), IncP-7 (MOB_H12_), and a number of others out of Inc assignment. Of course, results obtained by DPMT can help PBRT to design primers for the assessment of the newly discovered plasmid groups. As an example, the classification of virulence plasmids in the IncF and IncI1 complexes, reviewed by [26], will obviously gain by a joint PBRT+DPMT analysis.

An added advantage of the DPMT method is its applicability in the identification of ICEs (see figures 6 and 7). ICEs are also vehicles that disseminate virulence and AbR genes [66], [67]. They are known to constitute an integral part of most bacterial genomes, outnumbering plasmids by 2 to 1 in sequence databases [63]. ICEs are beginning to be closely linked to some of the more powerful AbR mechanisms such as ESBL, metallo- and AmpC type β-lactamases. For instance, chromosomal MOB_H121_ (R391-like) elements putatively involved in *bla*_CMY-2_ mobilization were detected by DPMT in enterobacterial isolates [64]. The MOB families considered in our primer set are also abundant in ICEs of γ-Proteobacteria [63]. The expanded diversity that DPMT discovered in γ-proteobacterial plasmids (and ICEs) will help to populate poorly solved branches of the existent phylogenetic trees and, therefore, lead to better consensus sequences to improve the design of new primer sets and, eventually, to design a multiplex set of non-degenerate oligonucleotides for faster plasmid screening and identification procedures (work in progress). Additionally, and due to their broad amplification capabilities, the DPMT method could be used in the analysis of plasmids and ICEs in total community DNA. In this case, the DNA fragments obtained from amplification with the 19 DPMT primer pairs could be combined and subjected to deep sequencing methodology. As a result, all amplifying sequences could be identified and quantified, resulting in a quantitative description of the plasmid and ICE composition of the analyzed populations and given environmental conditions.

The analysis of relaxases and replicons of γ-proteobacterial plasmids carried out in this and previous works strongly suggests that there is a high correlation between the MOB and the Inc/Rep group. That is, in a single MOB subfamily, relaxases from different Inc plasmids can be grouped, but plasmids of such Inc groups do not contain relaxases dispersed in different MOB subfamilies. Some exceptions are observed, which can usually be explained by plasmid cointegration and secondary deletions. Thus, DPMT provides not only the relaxase identity but a quick inference of the phylogenetic relationships with other plasmids as well as an idea of the constitution of the plasmid backbone. In summary, the combination of both methods, DPMT and PBRT, could better serve in the identification and characterization of plasmid species which are relevant in human and animal medicine. We hope they will help to inspire more effective clinical and environmental policies to manage the dreadful increase of more virulent and multi-antibiotic resistant human pathogens.

### Conclusions

The Degenerate Primer MOB Typing (DPMT) method allows rapid and accurate identification of transmissible plasmids based on their relaxase sequences. It detects a broader range of plasmids than the PCR-based replicon typing (PBRT) method and highlights a significant plasmid diversity that was underestimated. The DPMT method can be useful in the analysis of plasmids from both clinical and environmental isolates. The philosophy that guided the development of the γ-Proteobacteria MOB primer set can be easily extended to encompass relaxases of other taxonomical groups of bacteria.

## Methods

### Plasmids, Bacterial Strains, Growth Conditions and DNA Extraction

Relaxases representing five out of six MOB families described in Garcillán-Barcia, 2009 (MOB_F_, MOB_P_, MOB_Q_, MOB_H_, and MOB_C_) were used as standards for DPMT validation. MOB_V_ relaxases were not included since they are barely represented in γ-Proteobacteria. The resulting reference collection included six conjugative or mobilizable plasmids and 27 recombinant plasmids containing cloned relaxase genes (Table 1). For their construction, relaxase domains were delimited by using PSIpred (http://bioinf.cs.ucl.ac.uk/psipred/) [68], [69] and GOR (http://npsa-pbil.ibcp.fr/cgi-bin/npsa_automat.pl?page=npsa_gor4.html) [70]. Relaxase domains contained approximately the 300 N-terminal amino acids of these large multidomain proteins. Gene segments amplified by PCR were cloned either in the *Nde*I or *Nde*I/*Bam*HI sites of vector pET3a (Novagen) or in the *Nco*I/*Bam*HI sites of vector pET3d (Novagen), and introduced in *E. coli* DH5α by electroporation. Host strains were grown in Luria-Bertani broth (LB) in the presence of suitable antibiotics for plasmid selection. Total DNA was obtained using InstaGene Matrix (BioRad Laboratories), according to the manufactureŕs recommendations and starting from 100 µl saturated cultures.

### Bacterial Matings

Donors (*E. coli* primary isolates) and recipients (either DH5α [71] or HMS174 [72]) were grown to saturation, mixed in ratio 1∶1 and mated o/n on LB-agar plates at either 30°C or 37°C. Cells were resuspended in LB and dilutions plated on appropriate antibiotics (recipient marker + plasmid marker) to select for transconjugants. Nalidixic acid (20 µg/ml) was used to select for DH5α and rifampicin (50 µg/ml) for HMS174.

### Database Search

PSI-Blast [73] searches for relaxases were carried out using the N-terminal 300 amino acids of each MOB family prototype, following the method described in [36] and [35], but querying databases only for the subset of plasmids originally isolated from γ-Proteobacteria.

### PCR Primer Design

For each MOB family, relaxase domains were aligned and their phylogenetic relationships traced as previously described [35]. For each well-populated and well-resolved subfamily, the corresponding protein alignment was used to find blocks of at least four contiguous, usually invariant amino acids located within or close to the conserved relaxase motifs. Among them, two blocks were finally chosen to design forward and reverse primers for each subfamily. Oligonucleotide pairs were selected that detected most subfamily members while minimizing codon degeneracy and resulting in amplicons smaller than 400 bp. When a single primer pair did not encompass all subfamily members, it was further subdivided (*e.g.*, MOB_C1_ in C11 and C12). The primer pair for amplifying each MOB family was designed using CODEHOP [74] (Table 2). This strategy was already applied for the identification of DNA sequences of distantly related members of several gene families [75], [76], [77]. In CODEHOP, oligonucleotides derived from the selected blocks contain a 3′ partially-degenerate sequence, called CORE, comprising different codon variants of the highly conserved residues (11 nucleotides); and a 5′ non-degenerate sequence of variable size (around 14 nucleotides, to give a hybridization temperature of 55 to 60°C), called CLAMP, composed of the upstream contiguous nucleotides most conserved in the relaxase DNA alignment.

### Validation and Methodologies Comparison

Each primer pair was tested for amplification of the collection of 33 reference plasmids in standard PCR reactions. Each reaction contained PCR buffer (50mM KCl, 10 mM Tris-HCl (pH 8.8), 0.1% Triton X-100), 1.5 mM MgCl_2_, 0.2 mM dNTP, 1 µM of the corresponding pair of degenerate oligonucleotides, 2–5 µl (0.4–1 µg) of total DNA, and 1 U of BioTaq polymerase (Bioline) in a final volume of 50 µl. Details of amplification conditions for each primer pair are described in Table 2. Generally, the standard PCR protocol involved a 4 min step at 94°C, 25–30 cycles of 30 sec at 94°C, 30 sec at the annealing temperature and 30 sec at 72°C (the extension time had to be varied to adapt to the expected size of some amplicons; see Table 2 for details), and a final extension step for 10 min at 72°C. A touchdown PCR protocol [78] was used for amplification of MOB_H11_ and MOB_C11_ groups, to avoid the appearance of aberrant amplification products. It should be noted that the P4 primer pair (Table 2) fortuitously amplified a segment of some *Salmonella* chromosomes (corresponding to gene *fucO*, for instance in *S. typhimurium* DT104), thus impeding relaxase identification in this genomic background. No additional fortuitous amplicons were obtained when using clinical samples from *Escherichia*, *Salmonella* or *Klebsiella*. Amplicons were visualized after 2% agarose gel electrophoresis, using a GelDoc (BioRad Laboratories) and, when appropriate, sequenced by Macrogen Laboratories (Seoul, South Korea).

## Supporting Information

Table S1 **Plasmids from γ-Proteobacteria contained in the NCBI database.**
(DOC)

Information S1 **Nucleotide sequences and their translated amino acid sequences of relevant relaxases obtained by DPMT from different test collections.**
(DOC)
