# Supplementary material for: A Degenerate Primer MOB Typing (DPMT) Method to Classify Gamma-Proteobacterial Plasmids in Clinical and Environmental Settings
Source: PLoS One. 2012 Jul 11;7(7):e40438. doi: 10.1371/journal.pone.0040438 (PMC3394729; doi:10.1371/journal.pone.0040438)
Supplement: Table S1 — Plasmids from γ-Proteobacteria contained in the NCBI database. (DOC) [file pone.0040438.s001.doc]

# Table S1. Plasmids from γ-Proteobacteria contained in the NCBI database^1^.

| **GenBank ID** | **Plasmid** | | **Rep clasification^2^** | **MOB clasification** | | | **Host taxonomy^6^** | |
| --- | --- | --- | --- | --- | --- | --- | --- | --- |
|  | **Name** | **Size** |  | **MOB Class^3^** | **MOB sub-class^4^** | **DPMT ^5^** | **Original bacterial host** | **Family** |
| NC_007682 | pMUR050 | 56634 | YES; N_oriV_ [1]; N [2] | MOBF | F11 | YES (F11) | *Escherichia coli* | Enterobacteriaceae |
| NC_011383 | plasmid 9 | 70655 | YES; N_oriV_ [1] | MOBF | F11 | YES (F11) | *Klebsiella pneumoniae* | Enterobacteriaceae |
| NC_011385 | plasmid 12 | 75617 | YES; N_oriV_ [1]; N [2] | MOBF | F11 | YES (F11) | *Klebsiella pneumoniae* | Enterobacteriaceae |
| NC_010643 | R7K | 39792 | YES; W_oriV_ [1] ; W [2] | MOBF | F11 | YES (F11) | *Providencia rettgeri* | Enterobacteriaceae |
| NC_009982 | pMAK3 | 39924 | YES; W_oriV_ [1] ; W [2] | MOBF | F11 | YES (F11) | *Salmonella enterica* subsp. enterica serovar Dublin | Enterobacteriaceae |
| NC_010716 | pIE321 | 38150 | YES; W_oriV_ [1] ; W [2] | MOBF | F11 | YES (F11) | *Salmonella enterica* subsp. enterica serovar Dublin | Enterobacteriaceae |
| NC_009980 | pMAK2 | 61571 | YES; N_oriV_ [1]; N [2] | MOBF | F11 | YES (F11) | *Salmonella enterica* subsp. enterica serovar Dublin | Enterobacteriaceae |
| NC_011617 | pKP96 | 67850 | YES; N_oriV_ [1]; N [2] | MOBF | F11 | YES (F11) | *Klebsiella pneumoniae* | Enterobacteriaceae |
| NC_003292 | R46 | 50969 | YES; N_oriV_ [1]; N [2] | MOBF | F11 | YES (F11) | *Salmonella enterica* typhimurium | Enterobacteriaceae |
| NC_009132 | pLEW517 | 63946 | YES; N_oriV_ [1]; N [2] | MOBF | F11 | YES (F11) | *Escherichia coli* | Enterobacteriaceae |
| NC_009131 | pLEW517 | 65288 | YES; N_oriV_ [1]; N [2] | MOBF | F11 | YES (F11) | *Escherichia coli* | Enterobacteriaceae |
| NC_010891 | pCT14 | 55216 | NO | MOBF | F11 | YES (F11) | *Pseudomonas* sp. CT14 | Pseudomonadaceae |
| NC_007926 | NAH7 | 82232 | NO | MOBF | F11 | YES (F11) | *Pseudomonas putida* | Pseudomonadaceae |
| NC_003350 | pWW0 | 116580 | YES; P9_rep_ [3]; P9_korA3Fa-rep3Rc_; P9_ori3Fd-rep3Rc_ [4] | MOBF | F11 | YES (F11) | *Pseudomonas putida* | Pseudomonadaceae |
| NC_004999 | pDTG1 | 83042 | NO | MOBF | F11 | YES (F11) | *Pseudomonas putida* | Pseudomonadaceae |
| NC_007506 | pXCV38 | 38116 | NO | MOBF | F11 | YES (F11) | *Xanthomonas campestris* pv. vesicatoria str. 85-10 | Xanthomonadaceae |
| NC_005240 | pXcB | 37106 | NO | MOBF | F11 | YES (F11) | *Xanthomonas citri* | Xanthomonadaceae |
| NC_003922 | pXAC64 | 64920 | NO | MOBF | F11 | YES (F11) | *Xanthomonas axonopodis* pv. citri str. 306 | Xanthomonadaceae |
| NC_003277 | pSLT | 93939 | YES; FII_S_ [2]; FIB_S_ [5] | MOBF | F12 | YES (F12) | *Salmonella typhimurium* LT2 | Enterobacteriaceae |
| NC_010720 | p53638_75 | 75089 | YES; FrepB [2] | MOBF | F12 | YES (F12) | *Escherichia coli* 53638 | Enterobacteriaceae |
| NC_007208 | pOU1113 | 80156 | YES; FII_S_ [2] | MOBF | F12 | YES (F12) | *Salmonella enterica* | Enterobacteriaceae |
| NC_002483 | F | 99159 | YES; FIA, FIB, FIC [2]; FII [5] | MOBF | F12 | YES (F12) | *Escherichia coli* | Enterobacteriaceae |
| NC_011413 | pSE11-2 | 91158 | YES; FrepB [2] | MOBF | F12 | YES (F12) | *Escherichia coli* SE11 | Enterobacteriaceae |
| NC_009425 | pENTE01 | 157749 | NO | MOBF | F12 | YES (F12) | *Enterobacter* sp. 638 | Enterobacteriaceae |
| NC_011603 | pMAR2 | 97978 | YES; FrepB, FII_S_ [2] | MOBF | F12 | YES (F12) | *Escherichia coli* O127:H6 str. E2348/69 | Enterobacteriaceae |
| NC_011076 | pCVM29188_146 | 146811 | YES; FrepB, FIB [2] | MOBF | F12 | YES (F12) | *Salmonella enterica* subsp. enterica serovar Kentucky | Enterobacteriaceae |
| NC_010719 | p53638_226 | 225683 | YES; FrepB [2] | MOBF | F12 | YES (F12) | *Escherichia coli* 53638 | Enterobacteriaceae |
| NC_010409 | pVM01 | 151002 | YES; FrepB, FIB [2] | MOBF | F12 | YES (F12) | *Escherichia coli* | Enterobacteriaceae |
| NC_007675 | pAPEC-O2-ColV | 184501 | YES; FrepB, FIB [2] | MOBF | F12 | YES (F12) | *Escherichia coli* | Enterobacteriaceae |
| NC_006671 | pAPEC-O2-R | 101375 | YES; FrepB [2] | MOBF | F12 | YES (F12) | *Escherichia coli* | Enterobacteriaceae |
| NC_005327 | pC15-1a | 92353 | YES; FrepB [2] | MOBF | F12 | YES (F12) | *Escherichia coli* | Enterobacteriaceae |
| NC_006855 | pSCV50 | 49558 | YES; FIB, FII_S_ [2] | MOBF | F12 | YES (F12) | *Salmonella enterica* subsp. enterica serovar Cholerasuis | Enterobacteriaceae |
| NC_008460 | pO86A1 | 120730 | YES; FrepB, FIB [2] | MOBF | F12 | YES (F12) | *Escherichia coli* | Enterobacteriaceae |
| NC_010699 | pET45 | 44694 | NO | MOBF | F12 | YES (F12) | *Erwinia tasmaniensis* Et1/99 | Enterobacteriaceae |
| NC_010862 | pMAR7 | 101558 | YES; FrepB [2]; FIB_S_ [5] | MOBF | F12 | YES (F12) | *Escherichia coli* | Enterobacteriaceae |
| NC_009602 | pSFO157 | 121239 | YES; FrepB, FIB [2] | MOBF | F12 | YES (F12) | *Escherichia coli* | Enterobacteriaceae |
| NC_011812 | pO26I | 72946 | YES; FrepB [2] | MOBF | F12 | YES (F12) | *Escherichia coli* | Enterobacteriaceae |
| NC_009650 | pKPN4 | 107576 | YES; FII_K_ [5] | MOBF | F12 | YES (F12) | *Klebsiella pneumoniae* subsp. pneumoniae MGH | Enterobacteriaceae |
| NC_009837 | pAPEC-O1-ColBM | 174241 | YES; FrepB, FIB [2] | MOBF | F12 | YES (F12) | *Escherichia coli* APEC O1 | Enterobacteriaceae |
| NC_010558 | pIP1206 | 168113 | YES; FIA, FIB [2] | MOBF | F12 | YES (F12) | *Escherichia coli* 1520 | Enterobacteriaceae |
| NC_007941 | pUTI89 | 114230 | YES; FrepB, FIB [2] | MOBF | F12 | YES (F12) | *Escherichia coli* UTI89 | Enterobacteriaceae |
| NC_007385 | pSS_046 | 214396 | YES; FrepB [2] | MOBF | F12 | YES (F12) | *Shigella sonnei* Ss046 | Enterobacteriaceae |
| NC_010488 | pSMS35_135 | 130440 | YES; FrepB, FIB [2] | MOBF | F12 | YES (F12) | *Escherichia coli* SMS-3-5 | Enterobacteriaceae |
| NC_009133 | NR1 | 94289 | YES; FrepB [2] | MOBF | F12 | YES (F12) | *Escherichia coli* | Enterobacteriaceae |
| NC_009649 | pKPN3 | 175879 | YES; FII_K_ [5] | MOBF | F12 | YES (F12) | *Klebsiella pneumoniae* subsp. pneumoniae MGH | Enterobacteriaceae |
| NC_011747 | pECOS88 | 133853 | YES; FrepB, FIB [2] | MOBF | F12 | YES (F12) | *Escherichia coli* S88 | Enterobacteriaceae |
| NC_011749 | p1ESCUM | 122301 | YES; FrepB, FIB [2] | MOBF | F12 | YES (F12) | *Escherichia coli* UMN026 | Enterobacteriaceae |
| NC_004998 | p1658/97 | 125491 | YES; FIB [2]; FII [5] | MOBF | F12 | YES (F12) | *Escherichia coli* | Enterobacteriaceae |
| NC_010119 | pOU7519 | 127212 | YES; FIB_S_, FII_S_ [5] | MOBF | F12 | YES (F12) | *Salmonella enterica* subsp. enterica serovar Cholerasuis | Enterobacteriaceae |
| NC_002134 | R100 | 94281 | YES; FrepB [2] | MOBF | F12 | YES (F12) | *Shigella flexneri* | Enterobacteriaceae |
| NC_009702 | pC4602-1 | 56628 | NO | MOBF | F12 | YES (F12) | *Vibrio vulnificus* | Vibrionaceae |
| NC_010614 | pVT1 | 82266 | NO | MOBF | F12 | YES (F12) | *Vibrio tapetis* | Vibrionaceae |
| NC_005128 | pYJ016 | 48508 | NO | MOBF | F12 | YES (F12) | *Vibrio vulnificus* YJ016 | Vibrionaceae |
| NC_002638 | pKDSC50 | 49503 | YES; FIB_S_, FII_S_ [5] | MOBF,MOBF | F12 | YES (F12) | *Salmonella enterica* subsp. enterica serovar Cholerasuis | Enterobacteriaceae |
| NC_002698 | pWR501 | 221851 | YES; FII [5] | MOBF,MOBF | F12 | YES (F12) | *Shigella flexneri* | Enterobacteriaceae |
| NC_007607 | pSD1_197 | 182726 | YES; FrepB [2] | MOBF,MOBF | F12 | YES (F12) | *Shigella dysenteriae* Sd197 | Enterobacteriaceae |
| NC_007608 | pSB4_227 | 126697 | YES; FrepB [2] | MOBF,MOBF | F12 | YES (F12) | *Shigella boydii* Sb227 | Enterobacteriaceae |
| NC_004851 | pCP301 | 221618 | YES; RepAFII, FII [5] | MOBF,MOBF,MOBF | F12 | YES (F12) | *Shigella flexneri* 2a str. 301 | Enterobacteriaceae |
| NC_009352 | pAsal5 | 18361 | NO | MOBF | F12 | NO | *Aeromonas salmonicida* subsp. salmonicida | Aeromonadaceae |
| NC_009350 | pAsa5 | 155098 | NO | MOBF | F12 | NO | *Aeromonas salmonicida* subsp. salmonicida A449 | Aeromonadaceae |
| NC_006323 | pG8786 | 137036 | YES; FII_Y_ [5] | MOBF | F12 | NO | *Yersinia pestis* | Enterobacteriaceae |
| NC_009378 | MT | 137010 | YES; FII_Y_ [5] | MOBF | F12 | NO | *Yersinia pestis* str. Pestoides F | Enterobacteriaceae |
| NC_010606 | pACICU2 | 64366 | YES; GR6 [6] | MOBF | F12 | NO | *Acinetobacter baumannii* ACICU | Moraxellaceae |
| NC_010422 | pOU1115 | 74589 | YES; FII_S_ [5]; X1 [7] | MOBF,MOBP | F12, P3 | YES (P3) | *Salmonella enterica* subsp. enterica serovar Dublin | Enterobacteriaceae |
| NC_010377 | pYE854 | 95499 | NO | MOBF | NO | NO | *Yersinia enterocolitica* | Enterobacteriaceae |
| NC_006366 | pLPL | 59832 | NO | MOBF | NO | NO | *Legionella pneumophila* str. Lens | Legionellaceae |
| NC_006365 | pLPP | 131885 | NO | MOBF | NO | NO | *Legionella pneumophila* str. Paris | Legionellaceae |
| NC_011668 | pS22302 | 65448 | NO | MOBF | NO | NO | *Shewanella baltica* OS223 | Shewanellaceae |
| NC_011665 | pS22303 | 59223 | NO | MOBF | NO | NO | *Shewanella baltica* OS223 | Shewanellaceae |
| NC_011664 | pS22301 | 88311 | NO | MOBF | NO | NO | *Shewanella baltica* OS223 | Shewanellaceae |
| NC_009998 | pS19501 | 75605 | NO | MOBF | NO | NO | *Shewanella baltica* OS195 | Shewanellaceae |
| NC_009999 | pS19502 | 75508 | NO | MOBF | NO | NO | *Shewanella baltica* OS195 | Shewanellaceae |
| NC_009661 | pS18501 | 83224 | NO | MOBF | NO | NO | *Shewanella baltica* OS185 | Shewanellaceae |
| NC_005871 | pPBPR1 | 80033 | NO | MOBF | NO | NO | *Photobacterium profundum* SS9 | Vibrionaceae |
| NC_011311 | pVSAL840 | 83540 | NO | MOBF | NO | NO | *Aliivibrio salmonicida* LFI1238 | Vibrionaceae |
| NC_011204 | pCT02021853_74 | 74551 | YES; FII_S_ [5]; X1 [7] | MOBF,MOBP | NO, P3 | YES (P3) | *Salmonella enterica* subsp. enterica serovar Dublin | Enterobacteriaceae |
| NC_009704 | plasmid_59kb | 58679 | NO | MOBP | P1 | YES (P11) | *Yersinia pseudotuberculosis* IP 31758 | Enterobacteriaceae |
| NC_006388 | pB3 | 56167 | YES; P_trfA2_ [1]; trfA [8] | MOBP | P1 | YES (P11) | *Escherichia coli* | Enterobacteriaceae |
| NC_001735 | R751 | 53423 | YES; P_trfA2_ [1]; trfA [8] | MOBP | P1 | YES (P11) | *Enterobacter aerogenes* | Enterobacteriaceae |
| NC_009739 | pMATVIM-7 | 24179 | NO | MOBP | P1 | YES (P11) | *Pseudomonas aeruginosa* | Pseudomonadaceae |
| NC_008357 | pBS228 | 89147 | YES; P_trfA1_ [1]; P [2]; trfA [8] | MOBP | P1 | YES (P11) | *Pseudomonas aeruginosa* | Pseudomonadaceae |
| NC_010000 | pS19503 | 49148 | NO | MOBP | P1 | YES (P11) | *Shewanella baltica* OS195 | Shewanellaceae |
| NC_010579 | pXFAS01 | 38297 | NO | MOBP | P1 | YES (P11) | *Xylella fastidiosa* M23 | Xanthomonadaceae |
| NC_009788 | pETEC_73 | 70609 | YES; I1-Iγ [2] | MOBP | P1 | YES (P12) | *Escherichia coli* E24377A | Enterobacteriaceae |
| NC_011081 | pSL476_91 | 91374 | YES; I1-Iγ [2] | MOBP | P1 | YES (P12) | *Salmonella enterica* subsp*. enterica* serovar Heidelberg | Enterobacteriaceae |
| NC_002122 | ColIb-P9 | 93399 | YES; I1-Iγ [2] | MOBP | P1 | YES (P12) | *Escherichia coli* | Enterobacteriaceae |
| NC_011419 | pSE11-1 | 100021 | YES; I1-Iγ [2] | MOBP | P1 | YES (P12) | *Escherichia coli* SE11 | Enterobacteriaceae |
| NC_011078 | pCVM29188_46 | 46121 | NO | MOBP | P1 | YES (P12) | *Salmonella enterica* subsp*. enterica* serovar Kentucky | Enterobacteriaceae |
| NC_006856 | pSC138 | 138742 | NO | MOBP | P1 | YES (P12) | *Salmonella enterica* subsp*. enterica* serovar Cholerasuis | Enterobacteriaceae |
| NC_005014 | R64 | 120826 | YES; I1-Iγ [2] | MOBP | P1 | YES (P12) | *Salmonella typhimurium* | Enterobacteriaceae |
| NC_007365 | pO113 | 165548 | YES; FIB [2] | MOBP | P1 | YES (P12) | *Escherichia coli* | Enterobacteriaceae |
| NC_011754 | pECOED | 119594 | NO | MOBP | P1 | YES (P12) | *Escherichia coli* ED1a | Enterobacteriaceae |
| NC_011077 | pCVM29188_101 | 101461 | YES; I1-Iγ [2] | MOBP | P1 | YES (P12) | *Salmonella enterica* subsp*. enterica* serovar Kentucky | Enterobacteriaceae |
| NC_005246 | pEL60 | 60145 | YES; L/M [2] | MOBP | P1 | YES (P131) | *Erwinia amylovora* | Enterobacteriaceae |
| NC_004464 | pCTX-M3 | 89468 | YES; L/M [2] | MOBP | P1 | YES (P131) | *Citrobacter freundii* | Enterobacteriaceae |
| NC_011641 | pCTXM360 | 68018 | YES; L/M [2] | MOBP | P1 | YES (P131) | *Klebsiella pneumoniae* | Enterobacteriaceae |
| NC_005909 | pRA2 | 32743 | NO | MOBP | P1 | YES (P131) | *Pseudomonas alcaligenes* | Pseudomonadaceae |
| NC_010876 | pXAG81 | 26721 | NO | MOBP | P1 | YES (P131) | *Xanthomonas axonopodis* pv*. glycines* | Xanthomonadaceae |
| NC_004734 | pTC-F14 | 14155 | NO | MOBP | P1 | YES (P14) | *Acidithiobacillus caldus* | Acidithiobacillaceae |
| NC_003123 | pRAS3.1 | 11851 | NO | MOBP | P1 | YES (P14) | *Aeromonas salmonicida* subsp*. salmonicida* | Aeromonadaceae |
| NC_003124 | pRAS3.2 | 11823 | NO | MOBP | P1 | YES (P14) | *Aeromonas salmonicida* | Aeromonadaceae |
| NC_007100 | Rms149 | 57121 | NO | MOBP | P1 | YES (P14) | *Pseudomonas aeruginosa* | Pseudomonadaceae |
| NC_001520 | pTF4.1 | 4104 | NO | MOBP | P1 | NO | *Acidithiobacillus ferrooxidans* | Acidithiobacillaceae |
| NC_011207 | pBRST7.6 | 7621 | NO | MOBP | P1 | NO | *Aeromonas hydrophila* | Aeromonadaceae |
| NC_002128 | pO157 | 92721 | YES; FrepB, FIB [2] | MOBP | P1 | NO | *Escherichia coli* O157:H7 str. Sakai | Enterobacteriaceae |
| NC_008055 | QKH54 | 69966 | YES; trfA_γ [8] | MOBP | P1 | NO | *Escherichia coli* | Enterobacteriaceae |
| NC_010693 | pET46 | 46159 | NO | MOBP | P1 | NO | *Erwinia tasmaniensis* Et1/99 | Enterobacteriaceae |
| NC_002523 | pADAP | 153404 | NO | MOBP | P1 | NO | *Serratia entomophila* | Enterobacteriaceae |
| NC_007414 | pO157 | 92077 | YES; FrepB, FIB [2] | MOBP | P1 | NO | *Escherichia coli* O157:H7 EDL933 | Enterobacteriaceae |
| NC_011350 | pO157 | 94644 | YES; FrepB, FIB [2] | MOBP | P1 | NO | *Escherichia coli* O157:H7 str. EC4115 | Enterobacteriaceae |
| NC_007098 | pKMA2425 | 3156 | NO | MOBP | P1 | NO | *Actinobacillus pleuropneumoniae* | Pasteurellaceae |
| NC_004632 | pDC3000B | 67473 | NO | MOBP | P1 | NO | *Pseudomonas syringae* pv*. tomato* str. DC3000 | Pseudomonadaceae |
| NC_004633 | pDC3000A | 73661 | NO | MOBP | P1 | NO | *Pseudomonas syringae* pv*. tomato* str. DC3000 | Pseudomonadaceae |
| NC_010657 | pBS512_33 | 33103 | YES; X4 [7] | MOBP | P3 | YES (P3) | *Shigella boydii* CDC 3083-94 | Enterobacteriaceae |
| NC_010421 | pOU1114 | 34595 | YES; X1 [7] | MOBP | P3 | YES (P3) | *Salmonella enterica* subsp*. enterica* serovar Dublin | Enterobacteriaceae |
| NC_010860 | pSE34 | 32950 | YES; X1 [7] | MOBP | P3 | YES (P3) | *Salmonella enterica* subsp*. enterica* serovar Enteriditis | Enterobacteriaceae |
| NC_011739 | p2ESCUM | 33809 | YES; X1 [7] | MOBP | P3 | YES (P3) | *Escherichia coli* UMN026 | Enterobacteriaceae |
| NC_010555 | pHI4320 | 36289 | NO | MOBP | P3 | YES (P3) | *Proteus mirabilis* HI4320 | Enterobacteriaceae |
| NC_010378 | pOLA52 | 51602 | YES; X1 [7] | MOBP | P3 | YES (P3) | *Escherichia coli* | Enterobacteriaceae |
| NC_002579 | pVT745 | 25407 | NO | MOBP | P3 | YES (P3) | *Aggregatibacter actinomycetemcomitans* | Pasteurellaceae |
| NC_010919 | pRA3 | 45909 | YES; U [9] | MOBP | P4 | YES (P4) | *Aeromonas hydrophila* | Aeromonadaceae |
| NC_006143 | pFBAOT6 | 84749 | YES; U [9] | MOBP | P4 | YES (P4) | *Aeromonas punctata* | Aeromonadaceae |
| NC_002490 | pXF51 | 51158 | NO | MOBP | P4 | YES (P4) | *Xylella fastidiosa* 9a5c | Xanthomonadaceae |
| NC_004925 | pAsa2 | 5247 | NO | MOBP | P5 | YES (P51) | *Aeromonas salmonicida* subsp*. salmonicida* A449 | Aeromonadaceae |
| NC_004340 | pAsal3 | 5249 | NO | MOBP | P5 | YES (P51) | *Aeromonas salmonicida* subsp*. salmonicida* | Aeromonadaceae |
| NC_011407 | pSE11-4 | 6929 | NO | MOBP | P5 | YES (P51) | *Escherichia coli* SE11 | Enterobacteriaceae |
| NC_005910 | pAlvA | 5113 | NO | MOBP | P5 | YES (P51) | *Hafnia alvei* | Enterobacteriaceae |
| NC_005911 | pAlvB | 5216 | NO | MOBP | P5 | YES (P51) | *Hafnia alvei* | Enterobacteriaceae |
| NC_011082 | pSL476_3 | 3373 | NO | MOBP | P5 | YES (P51) | *Salmonella enterica* subsp*. enterica* serovar Heidelberg | Enterobacteriaceae |
| NC_011404 | pEC01 | 5002 | YES; ColE [9] | MOBP | P5 | YES (P51) | *Enterobacter cloacae* | Enterobacteriaceae |
| NC_009791 | pETEC_5 | 5033 | NO | MOBP | P5 | YES (P51) | *Escherichia coli* E24377A | Enterobacteriaceae |
| NC_009794 | pCKO2 | 5601 | NO | MOBP | P5 | YES (P51) | *Citrobacter koseri* ATCC BAA-895 | Enterobacteriaceae |
| NC_001371 | ColE1 | 6646 | NO | MOBP | P5 | YES (P51) | *Escherichia coli* | Enterobacteriaceae |
| NC_003457 | pC | 5269 | NO | MOBP | P5 | YES (P51) | *Salmonella enteritidis* | Enterobacteriaceae |
| NC_003456 | pK | 4245 | YES; ColE, ColE_Tp_ [9] | MOBP | P5 | YES (P51) | *Salmonella enteritidis* | Enterobacteriaceae |
| NC_011418 | pEC278 | 4669 | YES; ColE, ColE_Tp_ [9] | MOBP | P5 | YES (P51) | *Escherichia coli* | Enterobacteriaceae |
| NC_010898 | pIGRW12 | 4995 | YES; ColE [9] | MOBP | P5 | YES (P51) | *Escherichia coli* | Enterobacteriaceae |
| NC_011408 | pSE11-5 | 5366 | YES; ColE [9] | MOBP | P5 | YES (P51) | *Escherichia coli* SE11 | Enterobacteriaceae |
| NC_010993 | pST728/06-2 | 10107 | YES; ColE, ColE_Tp_ [9] | MOBP | P5 | YES (P51) | *Salmonella enterica* subsp*. enterica* serovar Typhimurium | Enterobacteriaceae |
| NC_006881 | pColK-K235 | 8318 | NO | MOBP | P5 | YES (P51) | *Escherichia coli* | Enterobacteriaceae |
| NC_005970 | pRK2 | 5360 | YES; ColE [9] | MOBP | P5 | YES (P51) | *Escherichia coli* | Enterobacteriaceae |
| NC_011799 | p5217 | 5217 | NO | MOBP | P5 | YES (P51) | *Escherichia coli* | Enterobacteriaceae |
| NC_009807 | pTPqnrS-1a | 10066 | YES; ColE, ColE_Tp_ [9] | MOBP | P5 | YES (P51) | *Salmonella enterica* subsp*. enterica* serovar Typhimurium | Enterobacteriaceae |
| NC_005019 | pLG13 | 6293 | NO | MOBP | P5 | YES (P51) | *Escherichia coli* | Enterobacteriaceae |
| NC_001848 | pBERT | 4656 | YES; ColE, ColE_Tp_ [9] | MOBP | P5 | YES (P51) | *Salmonella enterica* subsp*. enterica* serovar Berta | Enterobacteriaceae |
| NC_010796 | pRK10 | 4241 | YES; ColE, ColE_Tp_ [9] | MOBP | P5 | YES (P51) | *Serratia marcescens* | Enterobacteriaceae |
| NC_010485 | pSMS35_8 | 8909 | NO | MOBP | P5 | YES (P51) | *Escherichia coli* SMS-3-5 | Enterobacteriaceae |
| NC_011214 | pSL491_5 | 5880 | NO | MOBP | P5 | YES (P51) | *Salmonella enterica* subsp*. enterica* serovar Virchow | Enterobacteriaceae |
| NC_003079 | pSFD10 | 4091 | NO | MOBP | P5 | YES (P51) | *Salmonella choleraesuis* | Enterobacteriaceae |
| NC_010672 | pBS512_7 | 7437 | NO | MOBP | P5 | YES (P51) | *Shigella boydii* CDC 3083-94 | Enterobacteriaceae |
| NC_001373 | ColA | 6720 | NO | MOBP | P5 | YES (P51) | *Escherichia coli* | Enterobacteriaceae |
| NC_000923 | pRAY | 6076 | NO | MOBP,MOBP | P5 | YES (P51) | *Acinetobacter sp.* SUN | Moraxellaceae |
| NC_002637 | pMHSCS1 | 4992 | NO | MOBP | P5 | YES (P52) | *Mannheimia haemolytica* | Pasteurellaceae |
| NC_001774 | pIG1 | 5360 | NO | MOBP | P5 | YES (P52) | *Pasteurella multocida* | Pasteurellaceae |
| NC_006868 | pCCK647 | 5198 | NO | MOBP | P5 | YES (P52) | *Pasteurella multocida* | Pasteurellaceae |
| NC_009625 | pARD3079 | 4065 | NO | MOBP | P5 | YES (P52) | *Actinobacillus pleuropneumoniae* | Pasteurellaceae |
| NC_010069 | p9555 | 5673 | NO | MOBP | P5 | YES (P52) | *Actinobacillus pleuropneumoniae* | Pasteurellaceae |
| NC_007097 | pKM757 | 4556 | NO | MOBP | P5 | YES (P52) | *Actinobacillus porcitonsillarum* | Pasteurellaceae |
| NC_010042 | p9956 | 5674 | NO | MOBP | P5 | YES (P52) | *Actinobacillus pleuropneumoniae* | Pasteurellaceae |
| NC_010941 | ABB7_B | 4236 | NO | MOBP | P5 | YES (P52) | *Actinobacillus pleuropneumoniae* serovar 7 str. AP76 | Pasteurellaceae |
| NC_010940 | APP7_C | 3533 | NO | MOBP | P5 | YES (P52) | *Actinobacillus pleuropneumoniae* serovar 7 str. AP76 | Pasteurellaceae |
| NC_006976 | pCCK3259 | 5317 | NO | MOBP | P5 | YES (P52) | *Mannheimia haemolytica* | Pasteurellaceae |
| NC_007099 | pPSAS1522 | 4244 | NO | MOBP | P5 | YES (P52) | *Actinobacillus pleuropneumoniae* | Pasteurellaceae |
| NC_006828 | pHS-Tet | 5147 | NO | MOBP | P5 | YES (P52) | *Haemophilus parasuis* | Pasteurellaceae |
| NC_004338 | pAsal1 | 6371 | NO | MOBP | P5 | YES (P53) | *Aeromonas salmonicida* subsp*. salmonicida* | Aeromonadaceae |
| NC_004924 | pAsa3 | 5616 | NO | MOBP | P5 | YES (P53) | *Aeromonas salmonicida* subsp*. salmonicida* A449 | Aeromonadaceae |
| NC_004923 | pAsa1 | 5424 | NO | MOBP | P5 | YES (P53) | *Aeromonas salmonicida* subsp*. salmonicida* A449 | Aeromonadaceae |
| NC_004339 | pAsal2 | 5424 | NO | MOBP | P5 | YES (P53) | *Aeromonas salmonicida* subsp*. salmonicida* | Aeromonadaceae |
| NC_008488 | pMG828-3 | 5219 | NO | MOBP | P5 | NO | *Escherichia coli* | Enterobacteriaceae |
| NC_005325 | pEMCJH03 | 3510 | NO | MOBP | P5 | NO | *Moraxella catarrhalis* | Moraxellaceae |
| NC_009516 | pRWF101 | 13956 | NO | MOBP | P5 | NO | *Psychrobacter sp*. PRwf-1 | Moraxellaceae |
| NC_010900 | pMBO-2 | 27075 | NO | MOBP | P5 | NO | *Moraxella bovis* Epp63 | Moraxellaceae |
| NC_005247 | pEU30 | 30314 | NO | MOBP | P6 | NO | *Erwinia amylovora* | Enterobacteriaceae |
| NC_010697 | pET49 | 48751 | NO | MOBP | P6 | NO | *Erwinia tasmaniensis* Et1/99 | Enterobacteriaceae |
| NC_010696 | pET35 | 35494 | NO | MOBP | P6 | NO | *Erwinia tasmaniensis* Et1/99 | Enterobacteriaceae |
| NC_011148 | unnamed | 37978 | NO | MOBP | P6 | NO | *Salmonella enterica* subsp*. enterica* serovar Agona | Enterobacteriaceae |
| NC_002525 | R721 | 75582 | NO | MOBP | P6 | NO | *Escherichia coli* | Enterobacteriaceae |
| NC_011351 | pEC4115 | 37452 | NO | MOBP | P6 | NO | *Escherichia coli* O157:H7 str. EC4115 | Enterobacteriaceae |
| NC_007206 | pF1947 | 32628 | NO | MOBP | P6 | NO | *Haemophilus influenzae* biotype *aegyptius* | Pasteurellaceae |
| NC_004846 | pF3031 | 32433 | NO | MOBP | P6 | NO | *Haemophilus influenzae* biotype *aegyptius* | Pasteurellaceae |
| NC_004058 | pF3028 | 32379 | NO | MOBP | P6 | NO | *Haemophilus influenzae* biotype *aegyptius* | Pasteurellaceae |
| NC_005918 | pPMA4326A | 46697 | NO | MOBP | P6 | NO | *Pseudomonas syringae* pv. *maculicola* | Pseudomonadaceae |
| NC_005919 | pPMA4326B | 40110 | NO | MOBP | P6 | NO | *Pseudomonas syringae* pv. *maculicola* | Pseudomonadaceae |
| NC_005205 | pPSR1 | 72601 | NO | MOBP | P6 | NO | *Pseudomonas syringae* pv. *syringae* | Pseudomonadaceae |
| NC_007275 | Small plasmid | 51711 | NO | MOBP | P6 | NO | *Pseudomonas syringae* pv. *phaseolicola* 1448A | Pseudomonadaceae |
| NC_009777 | pVIBHAR | 89008 | NO | MOBP | P6 | NO | *Vibrio harveyi* ATCC BAA-1116 | Vibrionaceae |
| NC_006842 | pES100 | 45849 | NO | MOBP | P6 | NO | *Vibrio fischeri* ES114 | Vibrionaceae |
| NC_001898 | pUCD5000 | 5229 | NO | MOBP | NO | YES (P51) | *Pantoea citrea* | Enterobacteriaceae |
| NC_002632 | pPvu1 | 4675 | NO | MOBP | NO | NO | *Proteus vulgaris* | Enterobacteriaceae |
| NC_011604 | pWES-1 | 10908 | NO | MOBP | NO | NO | *Salmonella enterica* subsp*. enterica* serovar Westhampton | Enterobacteriaceae |
| NC_010893 | pMBO-1 | 44215 | NO | MOBP | NO | NO | *Moraxella bovis* Epp63 | Moraxellaceae |
| NC_011131 | pLQ510 | 12084 | NO | MOBP | NO | NO | *Moraxella catarrhalis* | Moraxellaceae |
| NC_004771 | pJR1 | 6792 | NO | MOBP | NO | NO | *Pasteurella multocida* | Pasteurellaceae |
| NC_010795 | pHB0503 | 15079 | NO | MOBP | NO | NO | *Actinobacillus pleuropneumoniae* | Pasteurellaceae |
| NC_010675 | pKW1 | 4583 | NO | MOBP | NO | NO | *Pseudoalteromonas* sp. 643A | Pseudoalteromonadaceae |
| NC_004961 | pPS41 | 6886 | NO | MOBP | NO | NO | *Vibrio* sp. 41 | Vibrionaceae |
| NC_010600 | pTcM1 | 65158 | NO | MOBP,MOBQ | NO | NO | *Acidithiobacillus caldus* | Acidithiobacillaceae |
| NC_002636 | DN1 | 5112 | NO | MOBQ | Q11 | YES (Q11) | *Dichelobacter nodosus* | Cardiobacteriaceae |
| NC_001740 | RSF1010 | 8684 | YES; Q_oriV_ [1] | MOBQ | Q11 | YES (Q11) | *Escherichia coli* | Enterobacteriaceae |
| NC_005312 | pMS260 | 8124 | NO | MOBQ | Q11 | YES (Q11) | *Actinobacillus pleuropneumoniae* | Pasteurellaceae |
| NC_011378 | pCCK1900 | 10226 | YES; Q_oriV_ [1] | MOBQ | Q11 | YES (Q11) | *Pasteurella multocida* | Pasteurellaceae |
| NC_006994 | pCCK381 | 10874 | NO | MOBV, MOBQ | NO, Q11 | YES (Q11) | *Pasteurella multocida* | Pasteurellaceae |
| NC_009781 | pIGJC156 | 5146 | NO | MOBQ | Q12 | YES (Q12) | *Escherichia coli* | Enterobacteriaceae |
| NC_011093 | pCVM19633_4 | 4585 | NO | MOBQ | Q12 | YES (Q12) | *Salmonella enterica* subsp. enterica sv. Schwarzengrund | Enterobacteriaceae |
| NC_003455 | pP | 4301 | NO | MOBQ | Q12 | YES (Q12) | *Salmonella enteritidis* | Enterobacteriaceae |
| NC_010659 | pBS512_5 | 5114 | NO | MOBQ | Q12 | YES (Q12) | *Shigella boydii* CDC 3083-94 | Enterobacteriaceae |
| NC_008489 | pMG828-4 | 7462 | NO | MOBQ | Q12 | YES (Q12) | *Escherichia coli* | Enterobacteriaceae |
| NC_010695 | pET09 | 9299 | NO | MOBQ | Q12 | YES (Q12) | *Erwinia tasmaniensis* Et1/99 | Enterobacteriaceae |
| NC_002056 | pSC101 | 9263 | NO | MOBQ | Q12 | YES (Q12) | *Salmonella typhimurium* | Enterobacteriaceae |
| NC_010310 | pAV2 | 15135 | NO | MOBQ | Q12 | YES (Q12) | *Acinetobacter venetianus* | Moraxellaceae |
| NC_011411 | pSE11-6 | 4082 | NO | MOBQ | Qu | YES (Qu) | *Escherichia coli* SE11 | Enterobacteriaceae |
| NC_010885 | pIGWZ12 | 4072 | NO | MOBQ | Qu | YES (Qu) | *Escherichia coli* | Enterobacteriaceae |
| NC_010883 | pIGMS5 | 6750 | NO | MOBQ | Qu | YES (Qu) | *Escherichia coli* | Enterobacteriaceae |
| NC_008487 | pMG828-2 | 4091 | NO | MOBQ | Qu | YES (Qu) | *Escherichia coli* | Enterobacteriaceae |
| NC_010486 | pSMS35_4 | 4074 | NO | MOBQ | Qu | YES (Qu) | *Escherichia coli* SMS-3-5 | Enterobacteriaceae |
| NC_011228 | pO26-S4 | 6758 | NO | MOBQ | Qu | YES (Qu) | *Escherichia coli* | Enterobacteriaceae |
| NC_007505 | pXCV19 | 19146 | NO | MOBQ | Qu | YES (Qu) | *Xanthomonas campestris* pv. vesicatoria str. 85-10 | Xanthomonadaceae |
| NC_009476 | pAb5S9 | 24716 | NO | MOBQ | Qu | NO | *Aeromonas bestiarum* | Aeromonadaceae |
| NC_010401 | p1ABAYE | 5644 | YES; GR11 [6] | MOBQ | Qu | NO | *Acinetobacter baumannii* AYE | Moraxellaceae |
| NC_009966 | pLD-TEX-KL | 66512 | NO | MOBQ | NO | NO | *Fluoribacter dumoffii* | Legionellaceae |
| NC_006877 | pMAC | 9540 | YES; GR8 [6] | MOBQ | NO | NO | *Acinetobacter baumannii* | Moraxellaceae |
| NC_010396 | p2ABSDF | 25014 | YES; GR12, GR18 [6] | MOBQ | NO | NO | *Acinetobacter baumannii* SDF | Moraxellaceae |
| NC_010398 | p3ABSDF | 24922 | YES; GR7, GR9, GR15 [6] | MOBQ | NO | NO | *Acinetobacter baumannii* SDF | Moraxellaceae |
| NC_010889 | p12494 | 14393 | NO | MOBQ | NO | NO | *Actinobacillus pleuropneumoniae* | Pasteurellaceae |
| NC_005921 | pPMA4326C | 8244 | NO | MOBQ | NO | NO | *Pseudomonas syringae* pv. maculicola | Pseudomonadaceae |
| NC_002305 | R27 | 180461 | YES; HI1 [2] | MOBH | H11 | YES (H11) | *Salmonella typhi* | Enterobacteriaceae |
| NC_003384 | pHCM1 | 218160 | YES; HI1 [2] | MOBH | H11 | YES (H11) | *Salmonella enterica* subsp. enterica serovar Typhi | Enterobacteriaceae |
| NC_009981 | pMAK1 | 208409 | YES; HI1 [2] | MOBH | H11 | YES (H11) | *Salmonella enterica* subsp. enterica serovar Cholerasuis | Enterobacteriaceae |
| NC_010870 | pK29 | 269674 | YES; HI2 [2] | MOBH | H11 | YES (H11) | *Klebsiella pneumoniae* | Enterobacteriaceae |
| NC_005211 | R478 | 274762 | YES; HI2 [2] | MOBH | H11 | YES (H11) | *Serratia marcescens* | Enterobacteriaceae |
| NC_009838 | pAPEC-O1-R | 241387 | NO | MOBH | H11 | YES (H11) | *Escherichia coli* APEC O1 | Enterobacteriaceae |
| NC_009349 | pAsa4 | 166749 | NO | MOBH | H12 | YES (H121) | *Aeromonas salmonicida* subsp. salmonicida A449 | Aeromonadaceae |
| NC_009141 | pIP1202 | 182913 | NO | MOBH | H12 | YES (H121) | *Yersinia pestis* biovar Orientalis str. IP275 | Enterobacteriaceae |
| NC_009140 | pSN254 | 176473 | NO | MOBH | H12 | YES (H121) | *Salmonella enterica* subsp. enterica serovar Newport | Enterobacteriaceae |
| NC_009139 | pYR1 | 158038 | NO | MOBH | H12 | YES (H121) | *Yersinia ruckeri* | Enterobacteriaceae |
| NC_008612 | pP99-018 | 150157 | NO | MOBH | H12 | YES (H121) | *Photobacterium damselae* subsp. piscicida | Vibrionaceae |
| NC_008613 | pP91278 | 131520 | NO | MOBH | H12 | YES (H121) | *Photobacterium damselae* subsp. piscicida | Vibrionaceae |
| NC_011838 | pCAR1.2 | 200231 | NO | MOBH | H12 | YES (H11) | *Pseudomonas resinovorans* | Pseudomonadaceae |
| NC_004444 | pCAR1 | 199035 | NO | MOBH | H12 | YES (H11) | *Pseudomonas resinovorans* | Pseudomonadaceae |
| NC_003905 | Rts1 | 217182 | YES; T [2] | MOBH | H12 | NO | *Proteus vulgaris* | Enterobacteriaceae |
| NC_008573 | plasmid 1 | 278942 | NO | MOBH | H12 | NO | *Shewanella* sp. ANA-3 | Shewanellaceae |
| NC_008739 | pMAQU02 | 213290 | NO | MOBH | H2 | YES (H2) | *Marinobacter aquaeolei* VT8 | Alteromonadaceae |
| NC_011409 | plasmid ICEhin1056 | 59393 | NO | MOBH | H2 | NO | *Haemophilus influenzae* | Pasteurellaceae |
| NC_009793 | pCKO3 | 9294 | NO | MOBC | C11 | YES (C11) | *Citrobacter koseri* ATCC BAA-895 | Enterobacteriaceae |
| NC_011382 | plasmid 15S | 23753 | NO | MOBC | C11 | YES (C11) | *Klebsiella pneumoniae* | Enterobacteriaceae |
| NC_002119 | CloDF13 | 9957 | NO | MOBC | C11 | YES (C11) | *Escherichia coli* | Enterobacteriaceae |
| NC_010726 | pMET-1 | 41723 | NO | MOBC | C12 | YES (C12) | *Klebsiella pneumoniae* | Enterobacteriaceae |
| NC_011092 | pCVM19633_110 | 110227 | NO | MOBC | C12 | YES (C12) | *Salmonella enterica* subsp. enterica serovar | Enterobacteriaceae |
| NC_006154 | pYptb32953 | 27702 | NO | MOBC | C12 | YES (C12) | *Yersinia pseudotuberculosis* IP 32953 | Enterobacteriaceae |
| NC_009779 | pESA2 | 31208 | NO | MOBC | C12 | YES (C12) | *Cronobacter sakazakii* ATCC BAA-894 | Enterobacteriaceae |
| NC_005814 | pCRY | 21742 | NO | MOBC | C12 | YES (C12) | *Yersinia pestis* biovar Microtus str. 91001 | Enterobacteriaceae |
| NC_010112 | p23023 | 52527 | NO | MOBC | C12 | YES (C12) | *Vibrio* sp. 23023 | Vibrionaceae |
| NC_006858 | pVS54 | 5360 | NO | MOBC | NO | NO | *Vibrio salmonicida* LFI1238 | Vibrionaceae |
| NC_011315 | pVSAL54 | 5360 | NO | MOBC | NO | NO | *Aliivibrio salmonicida* LFI1238 | Vibrionaceae |
| NC_011406 | pIGMS31 | 2520 | NO | MOBV | NO | NO | *Klebsiella pneumoniae* | Enterobacteriaceae |
| NC_005569 | pBHRK19 | 5721 | NO | MOBV | NO | NO | *Escherichia coli* | Enterobacteriaceae |
| NC_005568 | pBHRK18 | 5721 | NO | MOBV | NO | NO | *Escherichia coli* | Enterobacteriaceae |
| NC_009624 | pKMA202 | 13425 | NO | MOBV | NO | NO | *Actinobacillus porcitonsillarum* | Pasteurellaceae |
| NC_005920 | pPMA4326D | 4833 | NO | MOBV | NO | NO | *Pseudomonas syringae* pv. Maculicola | Pseudomonadaceae |
| NC_005922 | pPMA4326E | 4217 | NO | MOBV | NO | NO | *Pseudomonas syringae* pv. maculicola | Pseudomonadaceae |
| NC_005023 | pTF5 | 19792 | NO | NO | NO | NO | *Acidithiobacillus ferrooxidans* | Acidithiobacillaceae |
| NC_008738 | pMAQU01 | 239623 | NO | NO | NO | NO | *Marinobacter aquaeolei* VT8 | Alteromonadaceae |
| NC_007483 | plasmid A | 40420 | NO | NO | NO | NO | *Nitrosococcus oceani* ATCC 19707 | Chromatiaceae |
| NC_011526 | pQpRS_K_Q154 | 39280 | NO | NO | NO | NO | *Coxiella burnetii* CbuK_Q154 | Coxiellaceae |
| NC_010258 | QpRS | 39281 | NO | NO | NO | NO | *Coxiella burnetii* 'MSU Goat Q177' | Coxiellaceae |
| NC_009726 | pQpDG | 54179 | NO | NO | NO | NO | *Coxiella burnetii* Dugway 5J108-111 | Coxiellaceae |
| NC_004704 | pQpH1 | 37393 | NO | NO | NO | NO | *Coxiella burnetii* RSA 493 | Coxiellaceae |
| NC_002118 | QpH1 | 37329 | NO | NO | NO | NO | *Coxiella burnetii* | Coxiellaceae |
| NC_010115 | QpH1 | 37317 | NO | NO | NO | NO | *Coxiella burnetii* RSA 331 | Coxiellaceae |
| NC_002131 | QpDV | 32601 | NO | NO | NO | NO | *Coxiella burnetii* | Coxiellaceae |
| NC_009789 | pETEC_6 | 6199 | YES; ColE, ColE_Tp_ [9] | NO | NO | NO | *Escherichia coli* E24377A | Enterobacteriaceae |
| NC_009780 | pESA3 | 131196 | NO | NO | NO | NO | *Cronobacter sakazakii* ATCC BAA-894 | Enterobacteriaceae |
| NC_009787 | pETEC_35 | 34367 | YES; FrepB [2] | NO | NO | NO | *Escherichia coli* E24377A | Enterobacteriaceae |
| NC_009786 | pETEC_80 | 79237 | YES; FrepB [2] | NO | NO | NO | *Escherichia coli* E24377A | Enterobacteriaceae |
| NC_010635 | pYPTS01 | 69812 | YES; FII_Y_ [5] | NO | NO | NO | *Yersinia pseudotuberculosis* PB1/+ | Enterobacteriaceae |
| NC_002120 | pYVe227 | 69673 | YES; FII_Y_ [5] | NO | NO | NO | *Yersinia enterocolitica* | Enterobacteriaceae |
| NC_002127 | pOSAK1 | 3306 | NO | NO | NO | NO | *Escherichia coli* O157:H7 str. Sakai | Enterobacteriaceae |
| NC_006815 | pU302S | 3208 | NO | NO | NO | NO | *Salmonella typhimurium* | Enterobacteriaceae |
| NC_006816 | pU302L | 84514 | YES; FIA, FIB, P, [2] | NO | NO | NO | *Salmonella typhimurium* | Enterobacteriaceae |
| NC_011405 | pIGRK | 2348 | NO | NO | NO | NO | *Klebsiella pneumoniae* | Enterobacteriaceae |
| NC_009790 | pETEC_74 | 74224 | YES; FrepB [2] | NO | NO | NO | *Escherichia coli* E24377A | Enterobacteriaceae |
| NC_002809 | ColJs | 5210 | NO | NO | NO | NO | *Shigella sonnei* | Enterobacteriaceae |
| NC_010925 | pPA3.0 | 2901 | NO | NO | NO | NO | *Pantoea agglomerans* | Enterobacteriaceae |
| NC_001910 | pLeu-Sg | 7967 | NO | NO | NO | NO | *Buchnera aphidicola* (*Schizaphis graminum*) | Enterobacteriaceae |
| NC_001911 | pLeu-Dn | 7768 | NO | NO | NO | NO | *Buchnera aphidicola* | Enterobacteriaceae |
| NC_002487 | pCol-let | 5847 | NO | NO | NO | NO | *Escherichia coli* | Enterobacteriaceae |
| NC_011878 | pLeu-BCc | 6054 | NO | NO | NO | NO | *Buchnera aphidicola* (*Cinara cedri*) | Enterobacteriaceae |
| NC_011416 | pSE11-3 | 60555 | YES; FIB [2] | NO | NO | NO | *Escherichia coli* SE11 | Enterobacteriaceae |
| NC_011417 | pEC904 | 3066 | NO | NO | NO | NO | *Escherichia coli* | Enterobacteriaceae |
| NC_008122 | pCD | 70299 | YES; FII_Y_ [5] | NO | NO | NO | *Yersinia pestis* Antiqua | Enterobacteriaceae |
| NC_008120 | pMT | 96471 | NO | NO | NO | NO | *Yersinia pestis* Antiqua | Enterobacteriaceae |
| NC_008121 | pPCP | 10777 | NO | NO | NO | NO | *Yersinia pestis* Antiqua | Enterobacteriaceae |
| NC_010064 | pLMO226 | 2004 | NO | NO | NO | NO | *Escherichia coli* | Enterobacteriaceae |
| NC_010656 | pBS512_2 | 2089 | NO | NO | NO | NO | *Shigella boydii* CDC 3083-94 | Enterobacteriaceae |
| NC_008053 | pHW15 | 3002 | NO | NO | NO | NO | *Rahnella* sp. 'WMR15' | Enterobacteriaceae |
| NC_004429 | pIS2 | 6349 | NO | NO | NO | NO | *Escherichia coli* | Enterobacteriaceae |
| NC_008439 | pKKTET7 | 8401 | YES; ColE, ColE_Tp_ [9] | NO | NO | NO | *Shigella sonnei* | Enterobacteriaceae |
| NC_011266 | pO26-S1 | 1549 | NO | NO | NO | NO | *Escherichia coli* | Enterobacteriaceae |
| NC_004936 | pEsp1396 | 5622 | YES; ColE [9] | NO | NO | NO | *Enterobacter* sp. RFL1396 | Enterobacteriaceae |
| NC_002497 | pEI1 | 4807 | YES; ColE [9] | NO | NO | NO | *Edwardsiella ictaluri* | Enterobacteriaceae |
| NC_002498 | pEI2 | 5643 | NO | NO | NO | NO | *Edwardsiella ictaluri* | Enterobacteriaceae |
| NC_002144 | pYC | 5919 | NO | NO | NO | NO | *Yersinia pestis* | Enterobacteriaceae |
| NC_002145 | pKL1 | 1549 | NO | NO | NO | NO | *Escherichia coli* | Enterobacteriaceae |
| NC_002142 | pB171 | 68817 | YES; FIB_S_, FII [5] | NO | NO | NO | *Escherichia coli* | Enterobacteriaceae |
| NC_011281 | pKP91 | 91096 | YES; FII_K_ [5] | NO | NO | NO | *Klebsiella pneumoniae* 342 | Enterobacteriaceae |
| NC_011282 | pKP187 | 187922 | NO | NO | NO | NO | *Klebsiella pneumoniae* 342 | Enterobacteriaceae |
| NC_005570 | p29807 | 2682 | YES; ColE [9] | NO | NO | NO | *Yersinia enterocolitica* | Enterobacteriaceae |
| NC_001378 | pKYM | 2083 | NO | NO | NO | NO | *Shigella sonnei* | Enterobacteriaceae |
| NC_008597 | pVI678 | 6222 | YES; ColE, ColE_Tp_ [9] | NO | NO | NO | *Escherichia coli* | Enterobacteriaceae |
| NC_011602 | pE2348-2 | 6147 | YES; ColE, ColE_Tp_ [9] | NO | NO | NO | *Escherichia coli* O127:H6 str. E2348/69 | Enterobacteriaceae |
| NC_008119 | pPCP | 10778 | NO | NO | NO | NO | *Yersinia pestis* Nepal516 | Enterobacteriaceae |
| NC_008118 | pMT | 100918 | NO | NO | NO | NO | *Yersinia pestis* Nepal516 | Enterobacteriaceae |
| NC_008444 | p9705 | 3172 | NO | NO | NO | NO | *Escherichia coli* | Enterobacteriaceae |
| NC_011079 | pSL254_3 | 3605 | YES; ColE [9] | NO | NO | NO | *Salmonella enterica* subsp. enterica serovar Newport | Enterobacteriaceae |
| NC_010896 | pAnkS | 8271 | NO | NO | NO | NO | *Salmonella typhimurium* | Enterobacteriaceae |
| NC_010894 | pGY1 | 3592 | NO | NO | NO | NO | *Salmonella enterica* subsp. enterica serovar Paratyphi | Enterobacteriaceae |
| NC_002773 | p2457TS2 | 3179 | NO | NO | NO | NO | *Shigella flexneri* 2a | Enterobacteriaceae |
| NC_003789 | pMGD2 | 3564 | NO | NO | NO | NO | *Klebsiella* sp. KCL-2 | Enterobacteriaceae |
| NC_010257 | MccC7-H22 | 32014 | NO | NO | NO | NO | *Escherichia coli* | Enterobacteriaceae |
| NC_010259 | pA172 | 8197 | YES; ColE, ColE_Tp_ [9] | NO | NO | NO | *Salmonella enterica* subsp. enterica serovar Newport | Enterobacteriaceae |
| NC_009345 | pSS046_spA | 8401 | YES; ColE, ColE_Tp_ [9] | NO | NO | NO | *Shigella sonnei* Ss046 | Enterobacteriaceae |
| NC_003134 | pMT1 | 96210 | NO | NO | NO | NO | *Yersinia pestis* CO92 | Enterobacteriaceae |
| NC_005862 | Cryptic plasmid | 6066 | YES; ColE, ColE_Tp_ [9] | NO | NO | NO | *Salmonella enterica* subsp. enterica serovar | Enterobacteriaceae |
| NC_011227 | pO26-S3 | 4073 | NO | NO | NO | NO | *Escherichia coli* | Enterobacteriaceae |
| NC_003486 | pJHCMW1 | 11354 | YES; ColE, ColE_Tp_ [9] | NO | NO | NO | *Klebsiella pneumoniae* | Enterobacteriaceae |
| NC_010660 | pBS512_211 | 210919 | YES; FrepB [2] | NO | NO | NO | *Shigella boydii* CDC 3083-94 | Enterobacteriaceae |
| NC_005324 | p9123 | 6222 | YES; ColE, ColE_Tp_ [9] | NO | NO | NO | *Escherichia coli* | Enterobacteriaceae |
| NC_001537 | pECO29 | 3895 | NO | NO | NO | NO | *Escherichia coli* | Enterobacteriaceae |
| NC_008486 | pMG828-1 | 1902 | NO | NO | NO | NO | *Escherichia coli* | Enterobacteriaceae |
| NC_009705 | plasmid_153kb | 153140 | NO | NO | NO | NO | *Yersinia pseudotuberculosis* IP 31758 | Enterobacteriaceae |
| NC_010500 | pUO-SbR5 | 6478 | NO | NO | NO | NO | *Salmonella enterica* subsp. enterica serovar Brandenburg | Enterobacteriaceae |
| NC_005248 | pIGAL1 | 8145 | YES; ColE, ColE_Tp_ [9] | NO | NO | NO | *Escherichia coli* | Enterobacteriaceae |
| NC_005249 | pLVPK | 219385 | NO | NO | NO | NO | *Klebsiella pneumoniae* | Enterobacteriaceae |
| NC_008490 | pMG828-5 | 8985 | YES; ColE, ColE_Tp_ [9] | NO | NO | NO | *Escherichia coli* | Enterobacteriaceae |
| NC_010261 | pH205 | 8197 | YES; ColE, ColE_Tp_ [9] | NO | NO | NO | *Klebsiella pneumoniae* | Enterobacteriaceae |
| NC_011514 | pTN38148 | 10669 | NO | NO | NO | NO | *Escherichia coli* | Enterobacteriaceae |
| NC_011512 | p9701 | 4840 | NO | NO | NO | NO | *Klebsiella pneumoniae* | Enterobacteriaceae |
| NC_004564 | pYVa127/90 | 66591 | NO | NO | NO | NO | *Yersinia enterocolitica* | Enterobacteriaceae |
| NC_003114 | pCRP3 | 3172 | NO | NO | NO | NO | *Citrobacter rodentium* | Enterobacteriaceae |
| NC_004555 | pBBp1 | 2399 | NO | NO | NO | NO | *Buchnera aphidicola* str. Bp (Baizongia) | Enterobacteriaceae |
| NC_007635 | pCoo | 98396 | YES; FrepB, I1-Iγ [2] | NO | NO | NO | *Escherichia coli* | Enterobacteriaceae |
| NC_010886 | pK245 | 98264 | YES; R [9] | NO | NO | NO | *Klebsiella pneumoniae* | Enterobacteriaceae |
| NC_009716 | pAK51 | 6511 | NO | NO | NO | NO | *Escherichia* sp. Sflu5 | Enterobacteriaceae |
| NC_011795 | p6148 | 6148 | YES; ColE, ColE_Tp_ [9] | NO | NO | NO | *Escherichia coli* | Enterobacteriaceae |
| NC_010158 | pMT-pPCP | 114570 | NO | NO | NO | NO | *Yersinia pestis* Angola | Enterobacteriaceae |
| NC_010157 | new_pCD | 68190 | YES; FII_Y_ [5] | NO | NO | NO | *Yersinia pestis* Angola | Enterobacteriaceae |
| NC_005706 | pEA29 | 28185 | NO | NO | NO | NO | *Erwinia amylovora* | Enterobacteriaceae |
| NC_005018 | pKPN2 | 4196 | NO | NO | NO | NO | *Klebsiella pneumoniae* | Enterobacteriaceae |
| NC_005015 | pIP843 | 7086 | NO | NO | NO | NO | *Klebsiella pneumoniae* | Enterobacteriaceae |
| NC_005017 | pYVe8081 | 67720 | NO | NO | NO | NO | *Yersinia enterocolitica* | Enterobacteriaceae |
| NC_004940 | pEA1.7 | 1711 | NO | NO | NO | NO | *Erwinia amylovora* | Enterobacteriaceae |
| NC_004834 | pEJ30 | 29593 | NO | NO | NO | NO | *Erwinia* sp. Ejp 556 | Enterobacteriaceae |
| NC_004835 | pMT1 | 100984 | NO | NO | NO | NO | *Yersinia pestis* KIM | Enterobacteriaceae |
| NC_004836 | pCD1 | 70504 | YES; FII_Y_ [5] | NO | NO | NO | *Yersinia pestis* KIM | Enterobacteriaceae |
| NC_004837 | pPCP1 | 9610 | NO | NO | NO | NO | *Yersinia pestis* KIM | Enterobacteriaceae |
| NC_004838 | pMT-1 | 100990 | NO | NO | NO | NO | *Yersinia pestis* KIM | Enterobacteriaceae |
| NC_004839 | pCD1 | 70559 | YES; FII_Y_ [5] | NO | NO | NO | *Yersinia pestis* KIM | Enterobacteriaceae |
| NC_003131 | pCD1 | 70305 | YES; FII_Y_ [5] | NO | NO | NO | *Yersinia pestis* CO92 | Enterobacteriaceae |
| NC_003132 | pPCP1 | 9612 | NO | NO | NO | NO | *Yersinia pestis* CO92 | Enterobacteriaceae |
| NC_010499 | pUO-SbR3 | 6847 | YES; P [2] | NO | NO | NO | *Salmonella enterica* subsp. *enterica* serovar Brandenburg | Enterobacteriaceae |
| NC_007713 | pSG1 | 83306 | NO | NO | NO | NO | *Sodalis glossinidius* str. 'morsitans' | Enterobacteriaceae |
| NC_007714 | pSG2 | 27240 | NO | NO | NO | NO | *Sodalis glossinidius* str. 'morsitans' | Enterobacteriaceae |
| NC_007715 | pSG3 | 10810 | NO | NO | NO | NO | *Sodalis glossinidius* str. 'morsitans' | Enterobacteriaceae |
| NC_009829 | pSPRO01 | 46804 | NO | NO | NO | NO | *Serratia proteamaculans* 568 | Enterobacteriaceae |
| NC_006153 | pYV | 68525 | YES; FII_Y_ [5] | NO | NO | NO | *Yersinia pseudotuberculosis* IP 32953 | Enterobacteriaceae |
| NC_003435 | pCA4 | 3078 | NO | NO | NO | NO |  | Enterobacteriaceae |
| NC_005816 | pPCP1 | 9609 | NO | NO | NO | NO | *Yersinia pestis* biovar Microtus str. 91001 | Enterobacteriaceae |
| NC_002252 | pTrp | 7258 | NO | NO | NO | NO | *Buchnera aphidicola* str. APS | Enterobacteriaceae |
| NC_002253 | pLeu | 7786 | NO | NO | NO | NO | *Buchnera aphidicola* str. APS | Enterobacteriaceae |
| NC_002090 | NTP16 | 8255 | NO | NO | NO | NO | *Escherichia coli* | Enterobacteriaceae |
| NC_009653 | pKPN7 | 3478 | YES; ColE, ColE_Tp_ [9] | NO | NO | NO | *Klebsiella pneumoniae* subsp. pneumoniae MGH | Enterobacteriaceae |
| NC_009652 | pKPN6 | 4259 | YES; ColE, ColE_Tp_ [9] | NO | NO | NO | *Klebsiella pneumoniae* subsp. pneumoniae MGH | Enterobacteriaceae |
| NC_009651 | pKPN5 | 88582 | YES; R [9] | NO | NO | NO | *Klebsiella pneumoniae* subsp. pneumoniae MGH | Enterobacteriaceae |
| NC_009344 | pSD197_spA | 8953 | NO | NO | NO | NO | *Shigella dysenteriae* Sd197 | Enterobacteriaceae |
| NC_009347 | pSS046_spC | 2101 | NO | NO | NO | NO | *Shigella sonnei* Ss046 | Enterobacteriaceae |
| NC_009346 | pSS046_spB | 5153 | NO | NO | NO | NO | *Shigella sonnei* Ss046 | Enterobacteriaceae |
| NC_011767 | pECA1039 | 5620 | NO | NO | NO | NO | *Pectobacterium atrosepticum* | Enterobacteriaceae |
| NC_005002 | pB | 1983 | NO | NO | NO | NO | *Salmonella enteritidis* | Enterobacteriaceae |
| NC_004445 | pEP36 | 35904 | NO | NO | NO | NO | *Erwinia pyrifoliae* | Enterobacteriaceae |
| NC_004446 | pEA2.8 | 2825 | NO | NO | NO | NO | *Erwinia amylovora* | Enterobacteriaceae |
| NC_004843 | pBPS1 | 2308 | NO | NO | NO | NO | *Buchnera aphidicola* | Enterobacteriaceae |
| NC_010487 | pSMS35_3 | 3565 | YES; ColE [9] | NO | NO | NO | *Escherichia coli* SMS-3-5 | Enterobacteriaceae |
| NC_003425 | pWb1 | 5280 | NO | NO | NO | NO | *Wigglesworthia glossinidia* | Enterobacteriaceae |
| NC_005813 | pCD1 | 70159 | YES; FII_Y_ [5] | NO | NO | NO | *Yersinia pestis* biovar Microtus str. 91001 | Enterobacteriaceae |
| NC_005815 | pMT1 | 106642 | NO | NO | NO | NO | *Yersinia pestis* biovar Microtus str. 91001 | Enterobacteriaceae |
| NC_005923 | pFL129 | 6464 | NO | NO | NO | NO | *Escherichia coli* | Enterobacteriaceae |
| NC_011759 | pGDT4 | 94967 | NO | NO | NO | NO | *Yersinia pseudotuberculosis* | Enterobacteriaceae |
| NC_011752 | p55989p | 72482 | YES; FIB [2] | NO | NO | NO | *Escherichia coli* 55989 | Enterobacteriaceae |
| NC_003385 | pHCM2 | 106516 | NO | NO | NO | NO | *Salmonella enterica* subsp. enterica serovar Typhi | Enterobacteriaceae |
| NC_011215 | pSL491_3 | 3176 | NO | NO | NO | NO | *Salmonella enterica* subsp. enterica serovar Virchow | Enterobacteriaceae |
| NC_002070 | pPZG500 | 3661 | NO | NO | NO | NO | *Pantoea citrea* | Enterobacteriaceae |
| NC_010101 | pMM200 | 12245 | NO | NO | NO | NO | *Escherichia coli* | Enterobacteriaceae |
| NC_010100 | pMM234 | 9080 | NO | NO | NO | NO | *Escherichia coli* | Enterobacteriaceae |
| NC_011743 | pEFER | 55150 | YES; R [9] | NO | NO | NO | *Escherichia fergusonii* | Enterobacteriaceae |
| NC_002610 | pKlebB-k17/80 | 5258 | YES; ColE, ColE_Tp_ [9] | NO | NO | NO | *Klebsiella pneumoniae* | Enterobacteriaceae |
| NC_007183 | pSG1 | 81553 | NO | NO | NO | NO | *Sodalis glossinidius* | Enterobacteriaceae |
| NC_007182 | pSG1 | 81553 | NO | NO | NO | NO | *Sodalis glossinidius* | Enterobacteriaceae |
| NC_007185 | pSG2 | 27240 | NO | NO | NO | NO | *Sodalis glossinidius* | Enterobacteriaceae |
| NC_007184 | pSG2 | 27240 | NO | NO | NO | NO | *Sodalis glossinidius* | Enterobacteriaceae |
| NC_007187 | pSG4 | 10816 | NO | NO | NO | NO | *Sodalis glossinidius* | Enterobacteriaceae |
| NC_007186 | pSG3 | 19201 | NO | NO | NO | NO | *Sodalis glossinidius* | Enterobacteriaceae |
| NC_007188 | pSG4 | 10816 | NO | NO | NO | NO | *Sodalis glossinidius* | Enterobacteriaceae |
| NC_010731 | pSP70 | 3306 | NO | NO | NO | NO | *Escherichia coli* | Enterobacteriaceae |
| NC_009595 | pCD1 | 70305 | YES; FII_Y_ [5] | NO | NO | NO | *Yersinia pestis* CA88-4125 | Enterobacteriaceae |
| NC_009596 | pMT1 | 96210 | NO | NO | NO | NO | *Yersinia pestis* CA88-4125 | Enterobacteriaceae |
| NC_011511 | p169 | 3679 | NO | NO | NO | NO | *Klebsiella pneumoniae* | Enterobacteriaceae |
| NC_011513 | pRPCMY | 3805 | NO | NO | NO | NO | *Proteus mirabilis* | Enterobacteriaceae |
| NC_009377 | CD | 71507 | YES; FII_Y_ [5] | NO | NO | NO | *Yersinia pestis* Pestoides F | Enterobacteriaceae |
| NC_011422 | pBSSB1 | 27037 | NO | NO | NO | NO | *Salmonella enterica* subsp. enterica serovar Typhi | Enterobacteriaceae |
| NC_011640 | pKpn114 | 4211 | YES; ColE, ColE_Tp_ [9] | NO | NO | NO | *Klebsiella pneumoniae* | Enterobacteriaceae |
| NC_008791 | pYVe8081 | 67721 | NO | NO | NO | NO | *Yersinia enterocolitica* subsp. enterocolitica | Enterobacteriaceae |
| NC_010904 | pColG | 4715 | NO | NO | NO | NO | *Escherichia coli* | Enterobacteriaceae |
| NC_004989 | R478 | 2120 | NO | NO | NO | NO | *Serratia marcescens* | Enterobacteriaceae |
| NC_006625 | pK2044 | 224152 | NO | NO | NO | NO | *Klebsiella pneumoniae* NTUH-K2044 | Enterobacteriaceae |
| NC_004952 | pFNL10 | 3990 | NO | NO | NO | NO | *Francisella novicida* | Francisellaceae |
| NC_010331 | pFPHI01 | 3936 | NO | NO | NO | NO | *Francisella philomiragia* subsp. philomiragia | Francisellaceae |
| NC_002109 | pOM1 | 4442 | NO | NO | NO | NO | *Francisella tularensis* | Francisellaceae |
| NC_009084 | pAB2 | 11302 | YES; GR2 [6] | NO | NO | NO | *Acinetobacter baumannii* ATCC 17978 | Moraxellaceae |
| NC_009083 | pAB1 | 13408 | NO | NO | NO | NO | *Acinetobacter baumannii* ATCC 17978 | Moraxellaceae |
| NC_010395 | p1ABSDF | 6106 | YES; GR1 [6] | NO | NO | NO | *Acinetobacter baumannii* SDF | Moraxellaceae |
| NC_010402 | p2ABAYE | 9661 | YES; GR2 [6] | NO | NO | NO | *Acinetobacter baumannii* AYE | Moraxellaceae |
| NC_010403 | p4ABAYE | 2726 | YES; GR14 [6] | NO | NO | NO | *Acinetobacter baumannii* AYE | Moraxellaceae |
| NC_010404 | p3ABAYE | 94413 | YES; GR13 [6] | NO | NO | NO | *Acinetobacter baumannii* AYE | Moraxellaceae |
| NC_002760 | pAC450 | 4379 | NO | NO | NO | NO | *Acinetobacter* sp. EB104 | Moraxellaceae |
| NC_001316 | pTA144 Dw | 1313 | NO | NO | NO | NO | *Moraxella* sp. TA144 | Moraxellaceae |
| NC_001315 | pTA144 | 1921 | NO | NO | NO | NO | *Moraxella* sp. TA144 | Moraxellaceae |
| NC_011585 | pAB0057 | 8729 | YES; GR2 [6] | NO | NO | NO | *Acinetobacter baumannii* AB0057 | Moraxellaceae |
| NC_009517 | pRWF102 | 2117 | NO | NO | NO | NO | *Psychrobacter* sp. PRwf-1 | Moraxellaceae |
| NC_010309 | pAV1 | 10820 | NO | NO | NO | NO | *Acinetobacter venetianus* | Moraxellaceae |
| NC_010481 | pABIR | 29823 | YES; GR12 [6] | NO | NO | NO | *Acinetobacter baumannii* | Moraxellaceae |
| NC_007968 | plasmid 1 | 41221 | NO | NO | NO | NO | *Psychrobacter cryohalolentis* K5 | Moraxellaceae |
| NC_010605 | pACICU1 | 28279 | YES; GR2, GR10 [6] | NO | NO | NO | *Acinetobacter baumannii* ACICU | Moraxellaceae |
| NC_005915 | pKAP298 | 49112 | NO | NO | NO | NO | *Caedibacter taeniospiralis* | Order Thiotrichales |
| NC_010912 | pYMH5 | 5047 | NO | NO | NO | NO | *Avibacterium paragallinarum* | Pasteurellaceae |
| NC_009623 | pKMA5 | 9549 | NO | NO | NO | NO | *Actinobacillus porcitonsillarum* | Pasteurellaceae |
| NC_005329 | pNAD1 | 5568 | NO | NO | NO | NO | *Haemophilus ducreyi* | Pasteurellaceae |
| NC_007800 | pCCK13698 | 14969 | NO | NO | NO | NO | *Bibersteinia trehalosi* | Pasteurellaceae |
| NC_004345 | p9L | 1338 | NO | NO | NO | NO | *Histophilus somni* | Pasteurellaceae |
| NC_005245 | p250 | 6286 | NO | NO | NO | NO | *Avibacterium paragallinarum* | Pasteurellaceae |
| NC_007094 | pKMA505 | 8632 | NO | NO | NO | NO | *Actinobacillus porcitonsillarum* | Pasteurellaceae |
| NC_004772 | pJR2 | 5252 | NO | NO | NO | NO | *Pasteurella multocida* | Pasteurellaceae |
| NC_003125 | pTYM1 | 4242 | NO | NO | NO | NO | *Actinobacillus pleuropneumoniae* | Pasteurellaceae |
| NC_010942 | APP7_A | 5685 | NO | NO | NO | NO | *Actinobacillus pleuropneumoniae* serovar 7 str. AP76 | Pasteurellaceae |
| NC_002664 | p57/98 | 1065 | NO | NO | NO | NO | *Histophilus somni* | Pasteurellaceae |
| NC_007096 | pKMA1467 | 11115 | NO | NO | NO | NO | *Actinobacillus porcitonsillarum* | Pasteurellaceae |
| NC_007095 | pIMD50 | 8751 | NO | NO | NO | NO | *Actinobacillus porcitonsillarum* | Pasteurellaceae |
| NC_003411 | pMVSCS1 | 5621 | NO | NO | NO | NO | *Mannheimia varigena* | Pasteurellaceae |
| NC_006829 | pHS-Rec | 9462 | NO | NO | NO | NO | *Haemophilus parasuis* | Pasteurellaceae |
| NC_006298 | pHS129 | 5178 | NO | NO | NO | NO | *Haemophilus somnus* 129PT | Pasteurellaceae |
| NC_002175 | pMTS1 | 1995 | NO | NO | NO | NO | *Methylophaga thalassica* | Piscirickettsiaceae |
| NC_010722 | pMM1 | 2140 | NO | NO | NO | NO | *Pseudomonas aeruginosa* | Pseudomonadaceae |
| NC_002518 | pPP81 | 2534 | NO | NO | NO | NO | *Pseudomonas putida* | Pseudomonadaceae |
| NC_009444 | pQBR103 | 425094 | NO | NO | NO | NO | *Pseudomonas fluorescens* SBW25 | Pseudomonadaceae |
| NC_002759 | pFKN | 39554 | NO | NO | NO | NO | *Pseudomonas syringae* pv. maculicola | Pseudomonadaceae |
| NC_005244 | pND6-1 | 101858 | NO | NO | NO | NO | *Pseudomonas* sp. ND6 | Pseudomonadaceae |
| NC_008275 | pWW53 | 107929 | NO | NO | NO | NO | *Pseudomonas putida* | Pseudomonadaceae |
| NC_007274 | Large plasmid | 131950 | NO | NO | NO | NO | *Pseudomonas syringae* pv. phaseolicola 1448A | Pseudomonadaceae |
| NC_006988 | p47L | 3084 | NO | NO | NO | NO | *Pseudomonas* sp. S-47 | Pseudomonadaceae |
| NC_006989 | p47S | 1782 | NO | NO | NO | NO | *Pseudomonas* sp. S-47 | Pseudomonadaceae |
| NC_004969 | pPS1M3 | 3138 | NO | NO | NO | NO | *Pseudoalteromonas* sp. PS1M3 | Pseudomonadaceae |
| NC_004951 | pNI10 | 5128 | NO | NO | NO | NO | *Pseudomonas fulva* | Pseudomonadaceae |
| NC_005009 | pYQ39 | 2297 | NO | NO | NO | NO | *Pseudomonas putida* | Pseudomonadaceae |
| NC_003892 | pQBR55 | 5924 | NO | NO | NO | NO | *Pseudomonas* sp. SLT2001 | Pseudomonadaceae |
| NC_008320 | plasmid 1 | 6499 | NO | NO | NO | NO | *Shewanella* sp. MR-7 | Shewanellaceae |
| NC_004349 | pMR-1 | 161613 | NO | NO | NO | NO | *Shewanella oneidensis* MR-1 | Shewanellaceae |
| NC_009037 | pSbal03 | 16762 | NO | NO | NO | NO | *Shewanella baltica* OS155 | Shewanellaceae |
| NC_009036 | pSbal02 | 74000 | NO | NO | NO | NO | *Shewanella baltica* OS155 | Shewanellaceae |
| NC_009035 | pSbal01 | 116763 | NO | NO | NO | NO | *Shewanella baltica* OS155 | Shewanellaceae |
| NC_009038 | pSbal04 | 7995 | NO | NO | NO | NO | *Shewanella baltica* OS155 | Shewanellaceae |
| NC_010910 | pVCG4.1 | 2163 | NO | NO | NO | NO | *Vibrio cholerae* | Vibrionaceae |
| NC_011403 | pES213 | 5501 | NO | NO | NO | NO | *Vibrio fischeri* | Vibrionaceae |
| NC_006860 | pSIO1 | 4906 | NO | NO | NO | NO | *Vibrio cholerae* | Vibrionaceae |
| NC_006859 | pVS43 | 4327 | NO | NO | NO | NO | *Vibrio salmonicida* LFI1238 | Vibrionaceae |
| NC_010734 | pAK1 | 13415 | NO | NO | NO | NO | *Vibrio shilonii* | Vibrionaceae |
| NC_010733 | pSFn1 | 11237 | NO | NO | NO | NO | *Vibrio nigripulchritudo* | Vibrionaceae |
| NC_010899 | pVCG1.2 | 2357 | NO | NO | NO | NO | *Vibrio cholerae* | Vibrionaceae |
| NC_010897 | pVCG1.1 | 4439 | NO | NO | NO | NO | *Vibrio cholerae* | Vibrionaceae |
| NC_008690 | pTC68 | 7847 | NO | NO | NO | NO | *Vibrio* sp. TC68 | Vibrionaceae |
| NC_005250 | pJM1 | 65009 | NO | NO | NO | NO | *Listonella anguillarum* | Vibrionaceae |
| NC_009703 | pC4602-2 | 66946 | NO | NO | NO | NO | *Vibrio vulnificus* | Vibrionaceae |
| NC_011316 | pVSAL43 | 4327 | NO | NO | NO | NO | *Aliivibrio salmonicida* LFI1238 | Vibrionaceae |
| NC_011314 | pVSAL320 | 30807 | NO | NO | NO | NO | *Aliivibrio salmonicida* LFI1238 | Vibrionaceae |
| NC_002088 | pSA19 | 4839 | NO | NO | NO | NO | *Vibrio parahaemolyticus* | Vibrionaceae |
| NC_009351 | pLO2 | 7941 | NO | NO | NO | NO | *Listonella anguillarum* serovar O2 | Vibrionaceae |
| NC_011797 | pBD146 | 7472 | NO | NO | NO | NO | *Vibrio fluvialis* | Vibrionaceae |
| NC_011185 | pMJ100 | 179459 | NO | NO | NO | NO | *Vibrio fischeri* MJ11 | Vibrionaceae |
| NC_002473 | pO3K6 | 8784 | NO | NO | NO | NO | *Vibrio parahaemolyticus* | Vibrionaceae |
| NC_010114 | p09022A | 31036 | NO | NO | NO | NO | *Vibrio* sp. 09022 | Vibrionaceae |
| NC_010113 | p0908 | 81413 | NO | NO | NO | NO | *Vibrio* sp. 0908 | Vibrionaceae |
| NC_004982 | pTLC | 4719 | NO | NO | NO | NO | *Vibrio cholerae* | Vibrionaceae |
| NC_009701 | pR99 | 68446 | NO | NO | NO | NO | *Vibrio vulnificus* | Vibrionaceae |
| NC_007504 | pXCV2 | 1852 | NO | NO | NO | NO | *Xanthomonas campestris* pv. vesicatoria str. 85-10 | Xanthomonadaceae |
| NC_007507 | pXCV183 | 182572 | NO | NO | NO | NO | *Xanthomonas campestris* pv. vesicatoria str. 85-10 | Xanthomonadaceae |
| NC_002489 | pXF1.3 | 1286 | NO | NO | NO | NO | *Xylella fastidiosa* 9a5c | Xanthomonadaceae |
| NC_003921 | pXAC33 | 33700 | NO | NO | NO | NO | *Xanthomonas axonopodis* pv. citri str. 306 | Xanthomonadaceae |
| NC_010429 | pSH1 | 6867 | NO | NO | NO | NO | *Stenotrophomonas maltophilia* | Xanthomonadaceae |
| NC_010872 | pAG1 | 15143 | NO | NO | NO | NO | *Xanthomonas axonopodis* pv. glycines | Xanthomonadaceae |
| NC_004554 | pXFPD1.3 | 1346 | NO | NO | NO | NO | *Xylella fastidiosa* Temecula1 | Xanthomonadaceae |
| NC_010887 | pXAG82 | 1315 | NO | NO | NO | NO | *Xanthomonas axonopodis* pv. glycines | Xanthomonadaceae |
| NC_010464 | pSM76 | 2927 | NO | NO | NO | NO | *Stenotrophomonas maltophilia* | Xanthomonadaceae |
| NC_002092 | pXF868 | 1296 | NO | NO | NO | NO | *Xylella fastidiosa* | Xanthomonadaceae |
| NC_001383 | pNKH43 | 1471 | NO | NO | NO | NO | *Xanthomonas maltophilia* | Xanthomonadaceae |
| NC_004987 | pXV64 | 1851 | NO | NO | NO | NO | *Xanthomonas euvesicatoria* | Xanthomonadaceae |

**^1^**: The table lists all plasmids from [10] that were isolated from γ -Proteobacteria.

**^2^**: PBRTclassification. The oligonucleotides used in our theoretical exercise are those reported by [1], [3], [4], [2], [8], [9], [6], [5], [11] and [7]. A plasmid was considered amplifiable (“YES”) when the last 12 nucleotides of the 3’ end of both oligonucleotides of a primer pair were 100% identical to the template (this is a relaxed requirement; judging by our observations, more extensive homology to the templates is required for effective amplification). Otherwise, we assumed that no amplification will occur (represented by “NO”).

**^3^**: MOB family to which a relaxase belongs to. “NO” means that the plasmid lacks any relaxase.

**^4^**: MOB sub-family of a given relaxase, as explained in the text (Figures 1 to 7). “NO” means that the relaxase was not assigned to any phylogenetic sub-division.

**^5^**: Name of the oligonucleotide pair suitable for DPMT amplification. We asume amplification will occur (“YES”) when the degenerated core of each primer pair member has 100% identity with the corresponding DNA target. Thus, “NO” indicates that no amplification is theoretically expected with any DPMT primer pair.

**^6^**: Original bacterial host from which each plasmid was first isolated and its corresponding bacterial taxonomic family.

References:

1. Gotz A, Pukall R, Smit E, Tietze E, Prager R, et al. (1996) Detection and characterization of broad-host-range plasmids in environmental bacteria by PCR*.* Appl Environ Microbiol 62: 2621-2628.

2. Carattoli A, Bertini A, Villa L, Falbo V, Hopkins KL, et al. (2005) Identification of plasmids by PCR-based replicon typing*.* J Microbiol Methods 63: 219-228.

3. Greated A, Thomas CM (1999) A pair of PCR primers for IncP-9 plasmids*.* Microbiology 145 ( Pt 11): 3003-3004.

4. Krasowiak R, Smalla K, Sokolov S, Kosheleva I, Sevastyanovich Y, et al. (2002) PCR primers for detection and characterisation of IncP-9 plasmids*.* FEMS Microbiol Ecol 42: 217-225.

5. Villa L, Garcia-Fernandez A, Fortini D, Carattoli A (2010) Replicon sequence typing of IncF plasmids carrying virulence and resistance determinants*.* J Antimicrob Chemother 65: 2518-2529.

6. Bertini A, Poirel L, Mugnier PD, Villa L, Nordmann P, et al. (2010) Characterization and PCR-based replicon typing of resistance plasmids in Acinetobacter baumannii*.* Antimicrob Agents Chemother 54: 4168-4177.

7. Johnson TJ, Bielak EM, Fortini D, Hansen LH, Hasman H, et al. (2012) Expansion of the IncX plasmid family for improved identification and typing of novel plasmids in drug-resistant Enterobacteriaceae*.* Plasmid 68: 43-50.

8. Bahl MI, Burmolle M, Meisner A, Hansen LH, Sorensen SJ (2009) All IncP-1 plasmid subgroups, including the novel epsilon subgroup, are prevalent in the influent of a Danish wastewater treatment plant*.* Plasmid 62: 134-139.

9. Garcia-Fernandez A, Fortini D, Veldman K, Mevius D, Carattoli A (2009) Characterization of plasmids harbouring qnrS1, qnrB2 and qnrB19 genes in Salmonella*.* J Antimicrob Chemother 63: 274-281.

10. Smillie C, Garcillan-Barcia MP, Francia MV, Rocha EP, de la Cruz F (2010) Mobility of plasmids*.* Microbiol Mol Biol Rev 74: 434-452.

11. Heuer H, Binh CT, Jechalke S, Kopmann C, Zimmerling U, et al. (2012) IncP-1epsilon Plasmids are Important Vectors of Antibiotic Resistance Genes in Agricultural Systems: Diversification Driven by Class 1 Integron Gene Cassettes*.* Front Microbiol 3: 2.
