# Supplementary material for: A Degenerate Primer MOB Typing (DPMT) Method to Classify Gamma-Proteobacterial Plasmids in Clinical and Environmental Settings
Source: PLoS One. 2012 Jul 11;7(7):e40438. doi: 10.1371/journal.pone.0040438 (PMC3394729; doi:10.1371/journal.pone.0040438)
Supplement: Information S1 — Nucleotide sequences and their translated amino acid sequences of relevant relaxases obtained by DPMT from different test collections. (DOC) [file pone.0040438.s002.doc]

**Supplementary Material S2.** Nucleotide sequences and their translated amino acid sequences, of relevant relaxases obtained by DPMT from different test collections.

**NikB-like protein of plasmid pAA-A3201**. The clinical isolate containing this plasmid was obtained from a woman with urinary tract infection in Sweden [1]. A portion of the relaxase gene was amplified using *P12-f*+*P1-r* primer pair. This plasmid relaxase belongs to MOB_P1_ family, P12 subfamily. Its amino acid sequence is 80% identical to NikB protein of plasmid pO113 (GenBank Acc. no. AAQ17653).

**Nucleotide sequence**:

gcgaaagatgataccgatccggtgtttcattatattctgagctggccggcgcatgaaagcccgcgcccgggcgcggcgtttctgtggtgcggccataccctgaaaagcctggaactgagcaaacatcagtatgtggcggcggtgcataccgataccgataacctgcatattcat

**Encoded protein sequence**:

AKDDTDPVFHYILSWPAHESPRPGAAFLWCGHTLKSLELSKHQYVAAVHTDTDNLHIH

**MobA/MobL-like protein from plasmid pAA-A3488**. The clinical isolate containing this plasmid was obtained from a woman with urinary tract infection in Sweden [1]. A portion of the relaxase gene was amplified using *Qu-f*+*Qu-r* primer pair. This plasmid relaxase belongs to MOB_Q_ family, Qu subfamily. Its amino acid sequence is 72% identical to MobA/MobL protein of plasmid pSMS35_4 (GenBank Acc. no. YP_001739884).

**Nucleotide sequence**:

gcggcgtatcgcagcggcagcctgatgctggatgaacgcaccggcctgaccccggattatacccgcaaacgccgctgcggcagctgcgatctgaccccggcg

**Encoded protein sequence**:

AAYRSGSLMLDERTGLTPDYTRKRRCGSCDLTPA

**TraI-like protein from plasmid pAA-TC1-79a**. The clinical isolate containing this plasmid was obtained from a Spanish hospital in 2002 [2]. A portion of the relaxase gene was amplified using *F11-f*+*F1-r* primer pair. This plasmid relaxase belongs to MOB_F_ family, F11 subfamily. Its amino acid sequence is 80% identical to TraI protein of plasmid R46 (GenBank Acc. no. AAL13397).

**Nucleotide sequence**:

gatgattattatagcaaagatagcagctttaccgcgtggcatggccagggcgcggaagcgctgggcctgagcggcgaagtgagcagccagcgctttaaagaactgctggtgggcgaaattgatccgtttacccagatgaaacgcagcagcggcgatgcgaccaaagaacgcctgggctatgatctgcatttt

**Encoded protein sequence**:

DDYYSKDSSFTAWHGQGAEALGLSGEVSSQRFKELLVGEIDPFTQMKRSSGDATKERLGYDLHF

**TraI-like protein from plasmid pAA-TC2-33a**. The clinical isolate containing this plasmid was obtained from a Spanish hospital in 2004 [3]. A portion of the relaxase gene was amplified using *F11-f*+*F1-r* primer pair. This plasmid relaxase belongs to MOB_F_ family, F11 subfamily. Its amino acid sequence is 80% identical to TraI protein of plasmid R46 (GenBank Acc. no. AAL13397).

**Nucleotide sequence**:

gatgattattatagcaaagatagcagctttaccgcgtggcatggccagggcgcggaagcgctgggcctgagcggcgaagtgagcagccagcgctttaaagaactgctggtgggcgaaattgatccgtttacccagatgaaacgcagcagcggcgatgcgaccaaagaacgcctgggctatgatctgcatttt

**Encoded protein sequence**:

DDYYSKDSSFTAWHGQGAEALGLSGEVSSQRFKELLVGEIDPFTQMKRSSGDATKERLGYDLHF

**TraI-like protein of plasmid pAA-TC1-14a**. The clinical isolate containing this plasmid was obtained from a Spanish hospital, during the period 2002-2004 [4]. A portion of the relaxase gene was amplified using *F12-f*+*F1-r* primer pair. This plasmid relaxase belongs to MOB_F_ family, F12 subfamily. Its amino acid sequence is 86% identical to TraI protein of plasmid pKPN3 (GenBank Acc. no. YP_001338645).

**Nucleotide sequence**:

gataactattatgtgattggcagcatggatgaacgctgccagggcaaaggcgcggaagcgctgggcctggaaggcaaagtggataaacaggtgtttaccgaactgctgcagggcaaactgccggatggcagcgatctgacccgcattcaggatggcgtgaacaaacatcgcccgggctatgatctgaccttt

**Encoded protein sequence**:

DNYYVIGSMDERCQGKGAEALGLEGKVDKQVFTELLQGKLPDGSDLTRIQDGVNKHRPGYDLTF

References:

1. Ejrnaes K, Stegger M, Reisner A, Ferry S, Monsen T, et al. (2006) Characteristics of Escherichia coli causing persistence or relapse of urinary tract infections: phylogenetic groups, virulence factors and biofilm formation. Virulence 2: 528-537.

2. Oliver A, Coque TM, Alonso D, Valverde A, Baquero F, et al. (2005) CTX-M-10 linked to a phage-related element is widely disseminated among Enterobacteriaceae in a Spanish hospital. Antimicrob Agents Chemother 49: 1567-1571.

3. Valverde A, Canton R, Hawkey P, Pitout J, Nordmann P, et al. (2009) International Dissemination of Extended Spectrum Beta-Lactamase CTX-M-14 in Enterobacteriaceae isolates is mainly associated with the spread of IncK and IncF plasmids. CMI 15: 2.

4. Coque TM, Novais A, Carattoli A, Poirel L, Pitout J, et al. (2008) Dissemination of clonally related Escherichia coli strains expressing extended-spectrum beta-lactamase CTX-M-15. Emerg Infect Dis 14: 195-200.
